# Dopant‐Regulated Piezocatalysts Evoke Sonopiezoelectric and Enzymatic PANoptosis for Synergistic Cancer Therapy

**DOI:** 10.1002/advs.202500406

**Published:** 2025-03-08

**Authors:** Linhong Zhong, Xun Guo, Liming Deng, Xiaoting Wang, Hongye He, Nianhong Wu, Rui Tang, Liang Chen, Yu Chen, Pan Li

**Affiliations:** ^1^ Ultrasound Department of the Second Affiliated Hospital of Chongqing Medical University Chongqing Key Laboratory of Ultrasound Molecular Imaging Chongqing 400010 P. R. China; ^2^ The First Affiliated Hospital of Chongqing Medical University Chongqing 400010 P. R. China; ^3^ Materdicine Lab School of Life Sciences Shanghai University Shanghai 200444 P. R. China; ^4^ Shanghai Institute of Materdicine Shanghai 200051 P. R. China

**Keywords:** enzymatic activity, hafnium oxide, nanocatalytic therapy, PANoptosis, piezoelectric biomaterials

## Abstract

Piezocatalyst‐enabled sonopiezoelectric therapy offers noninvasive treatment with high spatiotemporal selectivity, yet existing piezocatalysts are limited by suboptimal efficacy, cancer cell resistance to oxidative stress, and biosafety concerns. Here, hafnia (HfO_2_), one of the only few FDA‐approved inorganic nanomaterials for clinical trials, is identified as a promising piezocatalyst with high translational potential for sonopiezoelectric and enzymatic PANoptosis‐boosted nanocatalytic therapy. Specifically, engineered transition metal‐substituted HfO_2_ nanocatalysts are synthesized to optimize piezoelectric and enzyme‐mimicking activities. Among these, Mn‐substituted HfO_2_ with a 20% Mn ratio (HMO) demonstrates superior performance in sono‐triggered reactive oxygen species generation, attributed to its reduced bandgap and increased oxygen vacancies. HMO also exhibits multiple enzyme‐mimicking activities, including peroxidase (POD), catalase (CAT), and glutathione peroxidase (GPx), amplifying oxidative stress through tumor‐specific catalytic reactions. These dual catalytic effects enable the activation of cancer cell PANoptosis to elicit a robust antitumor immune response. Biological evaluations show significant tumor suppression and antitumor immune responses by HMO‐mediated nanocatalytic therapy. Unlike utilizing the radiosensitization ability of HfO_2_ in the clinic, this work unveils the distinctive sonopiezoelectric effect and multienzymatic activities of HfO_2_‐based nanocatalysts for biomedical applications, holding the potential to overcome the challenges of radiation damage associated with radiotherapy.

## Graphical abstract

Engineered Mn‐substituted HfO_2_ (HMO) is identified with enhanced sonopiezoelectric effects and multienzymatic activities, promoting reactive oxygen species generation and amplifying oxidative stress in cancer cells. Upon ultrasound irradiation, HMO evokes cancer cell PANoptosis, thereby triggering robust antitumor immunity. Such dual‐functional HMO‐based nanocatalyst offers a distinctive strategy for PANoptosis‐enhanced cancer treatment.

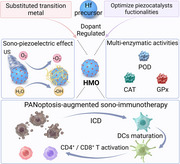

## 1 Introduction

Piezoelectric nanomaterials have attracted growing attention in a wide range of biomedical applications due to their unique ability to convert mechanical energy into electric energy.^[^ ^1^, ^2^, ^3^, ^4^, ^5^, ^6^, ^7^, ^8^, ^9^ ^]^ Ultrasound (US) can serve as a remotely controlled mechanical trigger to induce self‐polarization and the formation of a built‐in electrical field in piezoelectric nanocatalysts. Such a sonopiezoelectric process consistently separates electrons and holes, initiating surface redox reactions. The built‐in electric field facilitates efficient reactive oxygen species (ROS) generation by promoting the spontaneous migration of charge carriers to opposite directions within the crystal structure, opening new horizons for conventional sonodynamic therapy. Recently, a diversity of piezoelectric semiconductors, including BaTiO_3_, Bi_2_MoO_6_, ZnO, SnS, MoS_2_, KNbO_3_, Bi_4_Ti_3_O_12_, etc.,^[^ ^10^, ^11^, ^12^, ^13^, ^14^, ^15^, ^16^, ^17^, ^18^, ^19^, ^20^ ^]^ have been developed for therapeutic applications. Nevertheless, low ROS generation efficacy and undesirable biosafety profiles of most piezoelectric nanocatalysts remain major obstacles to their clinical translation. Despite several material engineering strategies that have been developed to enhance the piezocatalytic ROS generation, single‐mode piezocatalytic therapy often fails to achieve satisfactory therapeutic outcomes due to the inherent resistance of cancer cells against oxidative damage and the hypoxia tumor microenvironment. On the other hand, classical materials engineering strategies, such as constructing heterojunctions and defect engineering, might introduce heavy metal elements or compromise the colloidal stability, causing additional biosafety concerns.^[^ ^21^, ^22^ ^]^ To overcome the drawbacks mentioned above, there is an urgent need to develop new piezoelectric nanocatalysts with favorable biocompatibility and multifaceted catalytic activities.

While recent years have seen rapid advancements in piezoelectric nanocatalysts, the biomedical applications of functional nanomedicine, developed over several decades, continue to face significant challenges in achieving clinical success. Among the clinical candidates for cancer nanomedicine, hafnium (Hf)‐based nanomaterials have attracted considerable attention, particularly following the promising clinical trial outcomes of the nanosized radiosensitizer NBTXR3.^[^ ^23^, ^24^, ^25^, ^26^, ^27^, ^28^, ^29^ ^]^ NBTXR3, comprising a spherical HfO_2_ nanoparticle core with a negatively charged phosphate shell, significantly enhances ionizing radiation efficacy in radiotherapy due to the high atomic number of Hf. Completed phase II/III trials have demonstrated the superior efficacy of NBTXR3 compared with radiotherapy alone and their biosafety (ClinicalTrials. gov number: NCT01433068, NCT02379845, NCT01946867, NCT02721056). Additionally, several ongoing clinical trials are recruiting participants to evaluate the therapeutic efficacy of combining NBTXR3 with immunotherapy (NCT04892173, NCT05039632, NCT05039632). While HfO_2_ is one of the only approved biocompatible inorganic radiosensitizers for clinical trials, their sonopiezoelectric effects have not been explored.^[^ ^30^ ^]^ Additionally, the US offers a comparative tissue penetration depth yet superior biosafety with minimal side effects compared to traditional radiosensitization strategies. Recent first‐principles calculations have highlighted the intriguing piezoelectricity of nanoscale doped HfO_2_,^[^ ^31^, ^32^, ^33^ ^]^ yet experimental investigations into the biomedical potential of piezoelectric HfO_2_ nanomaterials remain underexplored. Given the ability of piezoelectric nanocatalysts to generate ROS in a spatiotemporally controlled manner under US stimulation, it is highly desirable to explore the sonopiezoelectric performance of biocompatible HfO_2_‐based nanocatalysts for versatile biomedical applications.

PANoptosis, an integrated form of programmed cell death (PCD) proposed in 2019,^[^ ^34^, ^35^ ^]^ involves the interactive activation processes and key features of pyroptosis, apoptosis, and necroptosis. Compared to other PCD pathways independently triggered by conventional cancer treatments, PANoptosis activation offers distinct advantages for immunotherapy by inducing a robust and sustained tumor‐specific immune response through extensive cellular damage and the continuous release of danger‐associated molecular patterns (DAMPs).^[^ ^36^, ^37^ ^]^ Whereas the intricate mechanisms of the PANoptosis pathway in cancer remain only partially understood, accumulating evidence suggests that DNA oxidative damage may act as an effective trigger for inducing PANoptosis.^[^ ^38^ ^]^ Piezoelectric nanocatalysts, which generate ROS within cancer cells under US stimulation, show significant promise for activating PANoptotic cell death. Nonetheless, challenges, such as the hypoxic tumor microenvironment and limited pro‐oxidative efficacy may impede this process. Hence, conferring enzyme‐mimicking capabilities to piezoelectric nanocatalysts is essential to overcoming these limitations and achieving effective PANoptosis‐sensitized immunotherapy.

**Scheme 1:**
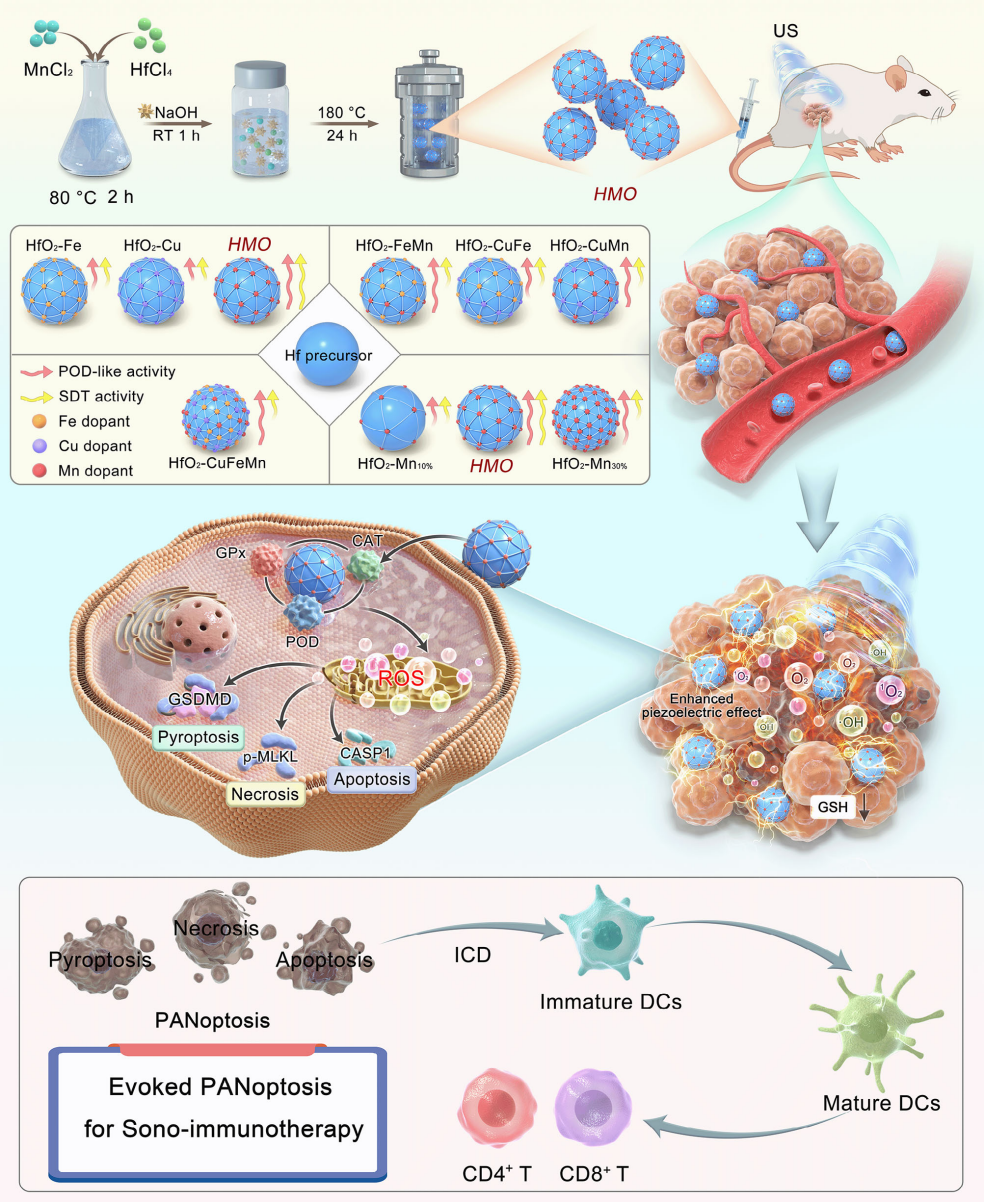
Schematic illustration of dopant‐regulated HMO sonopiezocatalytic/enzymatic nanocatalysts for nanocatalytic PANoptosis‐enhanced sono‐immunotherapy.

With the above‐mentioned considerations in mind, we propose a universal engineering strategy to synthesize a series of transition metals (Cu, Fe, Mn)‐substituted HfO_2_‐based nanocatalysts, with the aim of optimizing their bifunctional piezoelectric and enzymatic activities, for PANoptosis‐sensitized sono‐immunotherapy (**Scheme** **1**). Accordingly, single‐, double‐, and ternary‐doped HfO_2_ nanoparticles were synthesized, and their piezocatalytic effects under mechanical US stimulation, along with their enzyme‐mimicking activities, were comparatively evaluated. A notable correlation was observed between the doping composition and their sonopiezoelectric/enzymatic efficacy. Among all the evaluated nanocatalysts, rhombohedron‐shaped Mn‐doped HfO_2_ with a doping ratio of 20% (HMO) demonstrated the optimal balance of sonopiezoelectric ROS generation and enzyme‐mimicking activity. The enhanced piezoelectric response may be attributed to the smaller bandgap and increased oxygen vacancies after Mn substitution. Subsequently, HMO was selected to serve as a nanoactivator to induce the cancer cell PANoptosis for dual sonopiezoelectric/enzymatic nanocatalytic therapy. Compared to the currently developed inorganic piezocatalysts, the distinctive advantages of developed HMO lie in their certificated biosafety and enzymatically enhanced catalytic efficacy. Specifically, HMO facilitates the rapid production of singlet oxygen (^1^O_2_) through sonopiezoelectric effects, leading to oxidative damage in cancer cells. More importantly, the sonopiezoelectric therapeutic efficacy of HMO can be potentiated by their multiple enzyme‐mimicking activities of POD, GPx, and CAT under tumor microenvironment (TME). Benefiting from that, HMO upon US irradiation can effectively disrupt intracellular redox homeostasis, inducing PANoptosis in murine breast cancer cells (4T1) and eliciting an antitumor immune response through immunogenic cell death (ICD) induction. In vivo biological evaluations further revealed that HMO nanocatalysts achieved significant therapeutic efficacy in suppressing tumor growth. Given that both sono‐active and enzyme‐mimicking abilities of HfO_2_‐based nanomaterials have been scarcely explored, this study, to the best of our knowledge, presents the first paradigm of HfO_2_‐based nanocatalysts with sonopiezoelectric and multienzymatic activity for PANoptosis‐augmented sono‐immunotherapy.

## 2 Results and Discussion

### 2.1 Doping Optimization and Characterization of HMO Nanocatalysts

**Figure 1:**
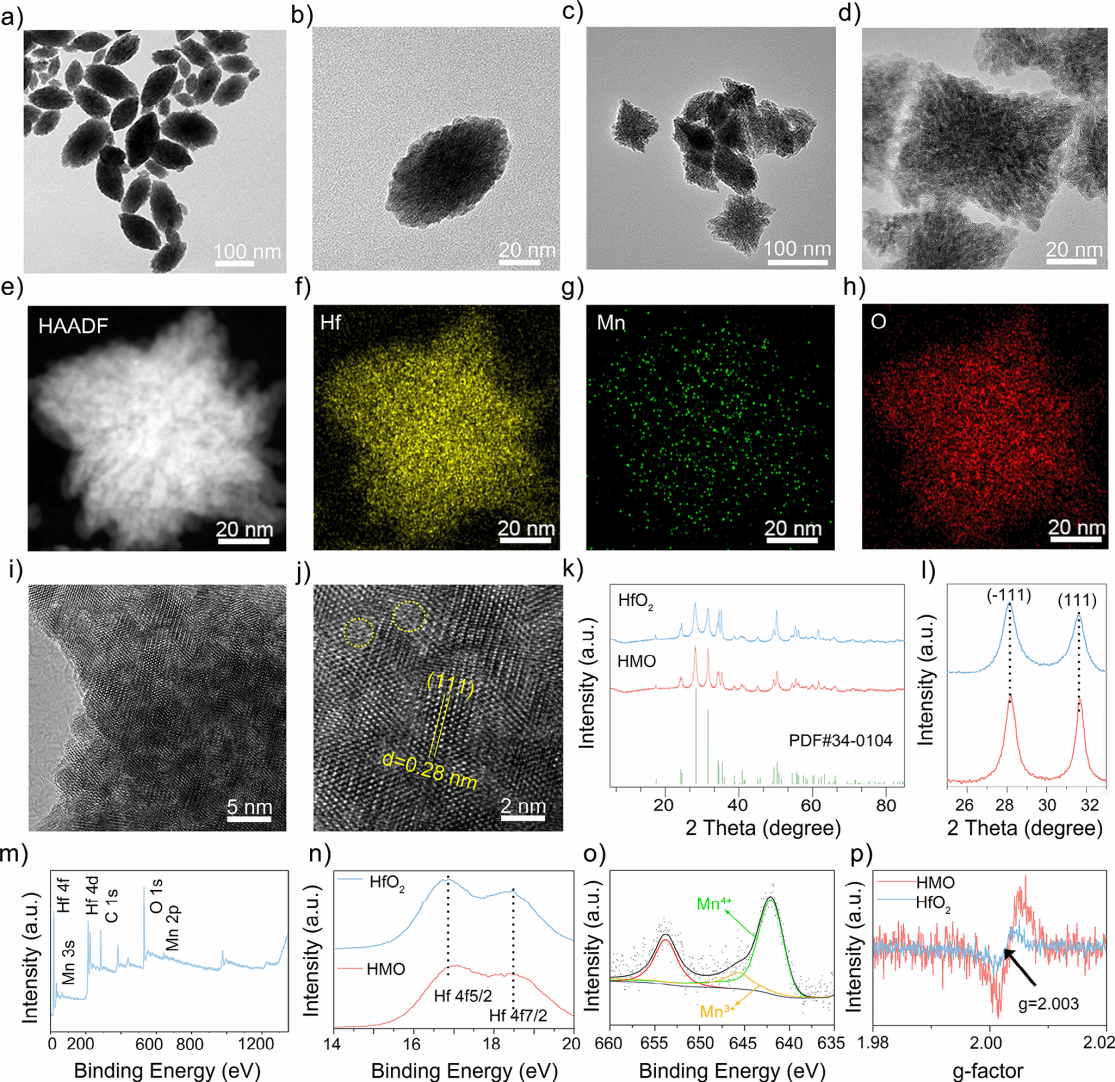
Morphology and characterization of HMO. a,b) Transmission electron microscope (TEM) images of HfO_2_. c,d) TEM images and e–h) Elemental mapping images of HMO. i,j) High‐resolution TEM images of HMO. k) X‐ray diffraction patterns of HfO_2_ and HMO, and l) partial enlargement for the XRD diffraction at (111) peaks. m) X‐ray photoelectron spectrum of HMO. n) Hf 4f spectrum for HMO, and o) Mn 2p spectrum for HMO. p) Electron paramagnetic resonance spectra of HfO_2_ and HMO.

Typically, transition metals‐substituted HfO_2_ nanocatalysts were synthesized by a facile hydrothermal method. We first selected MnCl_2_ and HfCl_4_ as the dopant and precursor, respectively. Transmission electron microscopy (TEM) images showed that undoped HfO_2_ nanoparticles exhibited a regular fusiform shape (**Figure** **1**a,b). In contrast, the morphology of HMO nanocatalysts, referred to HfO_2_ with 20% Mn doping, changed to irregular polyhedron (Figure 1c,d; and Figure S1a, Supporting Information). HMO nanocatalysts, referring to the sample with 20% Mn doping, appeared a relatively uniform particle size and a rhombohedron‐like nanoarchitecture. High‐angle annular dark‐field scanning transmission electron microscopy (HAADF‐STEM) images also revealed the uniform distribution of the Hf, Mn, and O elements within HMO (Figure 1e–h). The well‐defined nanoscale morphology and element distributions were also validated by scanning electron microscopy and energy‐dispersive X‐ray spectroscopy (Figure S2, Supporting Information). Moreover, high‐resolution TEM (HRTEM) images indicated a distinct lattice fringe with a crystal spacing of 0.28 nm, corresponding to the (111) diffraction plane of HMO (Figure 1i,j). Several disordered lattice defects were observed in the HRTEM image, likely attributed to the surface vacancies caused by the unsaturated coordination of metal atoms. The decrease in crystal spacing compared to dope‐free HfO_2_ nanoparticles is presumably ascribed to the incorporation of Mn atoms with a smaller atomic diameter into the lattices of HfO_2_ (Figure S3, Supporting Information). Furthermore, selected area electron diffraction (SAED) patterns showed a reduced number of diffraction rings in HMO nanocatalysts, signifying enlarged crystalline grains and improved crystallinity. In addition, the size distribution and zeta potential of HMO were investigated. Dynamic light scattering (DLS) results revealed that the hydrodynamic size of modified HMO remained almost unchanged in different solutions within a period of 48 h (Figure S1b–d, Supporting Information). Moreover, the zeta potentials of modified HMO in water, phosphate buffer solution (PBS), and fetal bovine serum (FBS) were determined to be about −25.2, −14.4, and −20.4 eV, respectively. These results evidence the favorable colloidal stability of modified HMO under the physiological environments.

Subsequently, the crystal structures of HfO_2_ before and after Mn doping were analyzed using X‐ray diffraction (XRD). As depicted in Figure 1k, both samples were characterized by a typical monoclinic phase with P21/a space group (JCPDS card no. 34‐0104). The absence of metallic Mn or oxide phases demonstrated that Mn atoms were successfully incorporated into the lattice of HfO_2_ without altering its phase structure. In the meantime, it was noteworthy that the diffraction peaks of Mn‐doped HfO_2_ slightly shifted to higher angles, indicating the shrinking of lattice space after Mn doping, which may contribute to the lattice distortion and increased polarity of HMO.^[^ ^21^ ^]^ The increased intensity and reduced width of peaks further corroborated the higher crystallinity of HMO than that of pure HfO_2_ nanoparticles (Figure 1l). X‐ray photoelectron spectroscopy (XPS) was employed to survey the surface chemical environments (Figure 1m). In the case of pure HfO_2_, the peaks located at 18.85 and 17.20 eV were assigned to 4f5/2 and 4f7/2 of Hf^4+^,^[^ ^39^ ^]^ respectively. After doping with Mn, the intensities of those two peaks for HMO decreased and shifted to higher binding energy (Figure 1n), suggesting that the electron density of Hf decreased owing to Mn doping.^[^ ^40^ ^]^ The high‐resolution XPS spectrum of Mn 2p3/2 exhibited two separated peaks at 640.5 and 642.0 eV (Figure 1o), corresponding to Mn^2+^ and Mn^3+^, respectively. The mixed valence state of Mn would theoretically endow HMO with enzyme‐like catalytic activity.^[^ ^41^ ^]^ More importantly, the existence of Mn^3+^ in the lattice of HMO suggested the nonequivalent substitution of Hf by Mn, thereby causing the formation of defects in the HfO_2_ lattice.^[^ ^42^ ^]^ Accordingly, the oxygen vacancies (OVs) in HMO nanocatalysts were further measured by electron paramagnetic resonance (EPR) analysis. As shown in Figure 1p, HMO displayed an intense signal at *g* = 2.003, corresponding to the electrons trapped on the OVs, whereas a weaker EPR signal was detected for HfO_2_.^[^ ^43^ ^]^ These OVs were expected to regulate the electronic structure and promote charge carriers, resulting in enhanced catalytic performance of HMO.

To extend the applicability of the doping strategy, the concentration and composition of the doping precursors were varied. The Mn‐substituted HfO₂ maintained its rhombohedral morphology even as the doping content increased to 30%, and no significant changes were observed in their crystal structures (Figure S4, Supporting Information). However, at a feeding ratio of 50% Mn precursor, both the nanoarchitecture and crystal structure were significantly disrupted, highlighting the critical impact of doping concentration. Fe‐ and Cu‐based precursors were also used to prepare single‐, binary‐, and ternary‐doped HfO_2_ nanostructures with a total doping content of 30%. Notably, all the doped HfO_2_ exhibited well‐defined nanostructures (Figure S5, Supporting Information). Moreover, strong diffraction peaks were detected in XRD patterns, implying the maintained crystallinity of doped HfO_2_ nanocatalysts. Element mapping images further validated the presence of corresponding metal elements within the nanostructures (Figure S6, Supporting Information). These observations solidify the assertion that such a mild hydrothermal method enables the flexible doping of various metal elements into HfO_2_, obtaining a bunch of biocompatible piezoelectric nanocatalysts.

### 2.2 SonoPiezoelectric Catalytic Performance of HMO Nanocatalysts

**Figure 2:**
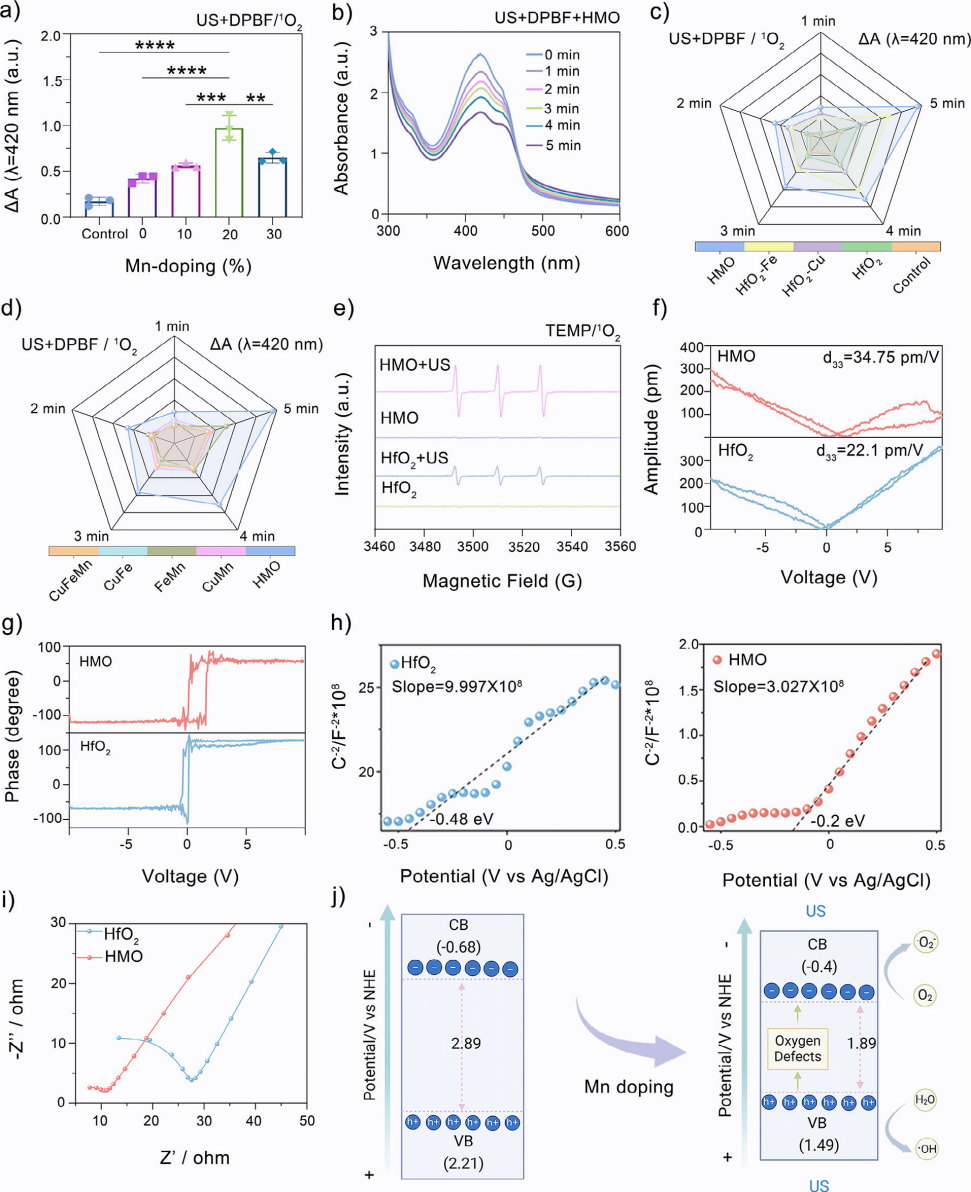
Sonopiezoelectric catalytic performance of HMO nanocatalysts. a) UV–vis absorbance changes of DPBF at 420 nm after incubated with the Mn‐substituted HfO_2_ at different Mn‐doping content under US excitation. Data are presented as mean ± standard deviation (S.D.) (*n* = 3). b) US‐triggered (1.0 MHz, 1.0 W cm^−2^, 50% cycle) ^1^O_2_ generation catalyzed by HMO. c) UV–vis absorbance changes of DPBF with different single metal‐ions doped HfO_2_ under US irradiation for different times. d) UV–vis absorbance changes of DPBF with different metal‐ion doped HfO_2_ by US triggered at different time. e) Electron spin resonance (ESR) spectra of TEMP/^1^O_2_ for the HfO_2_ and HMO with different treatments. f) Amplitude‐voltage curves and g) phase hysteresis loops of HfO_2_ and HMO. h) Mott–Schottky curves of HfO_2_ and HMO. i) Electrochemical impedance spectroscopy (EIS) curves of HfO_2_ and HMO. j) Schematic illustrations on the bandgap structure and catalytic mechanism for ROS generation capability of HMO under US irradiation. Created with BioRender.com.

The incorporation of transition metal elements in HfO_2_ is expected to endow the engineered nanocatalysts with dual sonopiezoelectric/enzymatic catalytic effects. First, to identify nanocatalysts with optimal sonopiezoelectric performance, we systematically evaluated the singlet oxygen (^1^O_2_) generation capacity of pure and diverse doped HfO_2_ nanocatalysts under US irradiation, using 1,3‐diphenylisobenzofuran (DPBF) as a fluorescent probe (Figure S7, Supporting Information). The results revealed that the sonopiezoelectric efficacy of Mn‐substituted HfO_2_ depended on the doping concentration, with the highest sono‐triggered ^1^O_2_ yield achieved in HMO (**Figure** **2**a,b). On the other hand, a majority of single metal‐doped samples exhibited negligible changes in the characteristic absorbance of DPBF at 420 nm under US irradiation (Figure 2c), suggesting their limited effectiveness in sonopiezoelectric catalytic ^1^O_2_ generation. Additionally, only FeMn‐doped samples showed a notable decrease in absorbance, while other binary and ternary metal‐doped HfO_2_ displayed minimal sonopiezoelectric ^1^O_2_ generation (Figure 2d). Therefore, HMO with 20% Mn doping was identified as an effective nanocatalyst with ROS generation capability, which presumably resulted from the sonopiezoelectric effects of HfO_2_. Electron spin resonance (ESR) measurements were performed to further verify the ^1^O_2_ generation capacity of HMO upon US irradiation, using 2,2,6,6‐tetramethylpiperidine (TEMP) as a ^1^O_2_ trapping agent. As expected, the US + HMO group exhibited typical 1:1:1 triplet signals (Figure 2e), with a higher intensity than that observed in nondoped HfO_2_.^[^ ^44^ ^]^ These findings validate the hypothesis that the metal‐substitution strategy can enhance the sonopiezoelectric efficacy of HfO_2_ nanocatalysts.

Next, the piezoelectricity of HfO_2_ and HMO was directly measured by piezoresponse force microscopy (PFM). A typical butterfly amplitude loop and ≈180° phase switching, a typical characteristic of piezoelectric materials, were observed when a bias voltage from −10 to 10 V was applied (Figure 2f,g).^[^ ^45^ ^]^ Notably, the amplitude variation of HMO was higher than that of HfO_2_, suggesting their stronger piezoelectric response. The piezoelectric coefficient (*d*_33_) is a key characteristic of the piezoelectric material, which reflects the conversion efficiency from mechanical energy to electricity.^[^ ^46^ ^]^ Here, the *d*_33_ of HMO was calculated to be ≈34.75 pm V^−1^ based on the amplitude‐voltage loop, which was ≈1.5 times higher than that of HfO_2_ (≈22.1 pm V^−1^), confirming the superior piezoelectric performance of HMO. As previous reports claimed that the piezoelectric effects of HfO_2_ were phase‐dependent,^[^ ^47^ ^]^ it is deduced that the piezoresponse of HMO may induced by the metal doping and resulting OVs.

To gain more insight into the catalytic mechanisms, Mott–Schottky curves of HfO_2_ and HMO were measured. The positive slope of the Mott–Schottky curves revealed the n‐type semiconductor of HfO_2_ and HMO (Figure 2h). To note, HMO showed a significantly smaller slope than that of HfO_2_, revealing a higher charge carrier density.^[^ ^48^ ^]^ Furthermore, we used electrochemical impedance spectroscopy (EIS) to characterize the separation efficiency of charge carriers in HfO_2_ and HMO. A smaller diameter of the semicircle was observed in HMO compared to the nondoped HfO_2_ (Figure 2i). This tendency corroborates the lower charge transfer resistance of the doped nanocatalysts, facilitating the separation of charge carriers upon US irradiation.^[^ ^49^ ^]^ Subsequently, the bandgaps of HfO_2_ and HMO were determined using the Kubelka–Munk (KM) function in the Tauc plot. The bandgap of HMO was calculated to be only 1.89 eV (Figure S8, Supporting Information), which is markedly narrower than that of HfO_2_ (≈2.89 eV), indicating that the impact of Mn doping in reducing the bandgap of HfO_2_. According to these observations, the catalytic mechanism for the ROS generation capability of HMO was proposed (Figure 2j). Based on the energy band level, the US can efficiently activate HMO nanocatalysts, exciting electrons from the valence band (VB) to the conduction band (CB) and creating holes in the VB. The separation of these electron–hole pairs is facilitated by the built‐in electric field, promoting the surface redox reaction to produce ¹O₂ and ·OH. Mn doping improves carrier transport by enhancing piezoelectric responses and generating a stronger internal electric field through lattice distortion and charge redistribution. This built‐in field promotes charge carrier separation and inhibits their recombination. Additionally, OVs in HMO can trap electrons, boosting carrier density and further enhancing catalytic efficiency. Taken together, HMO with optimized 20% Mn doping can acquire satisfactory piezoelectricity for efficient sonopiezoelctric ROS production.^[^ ^50^ ^]^

### 2.3 Multiple Enzyme‐Mimicking Activities of HMO Nanocatalysts

**Figure 3:**
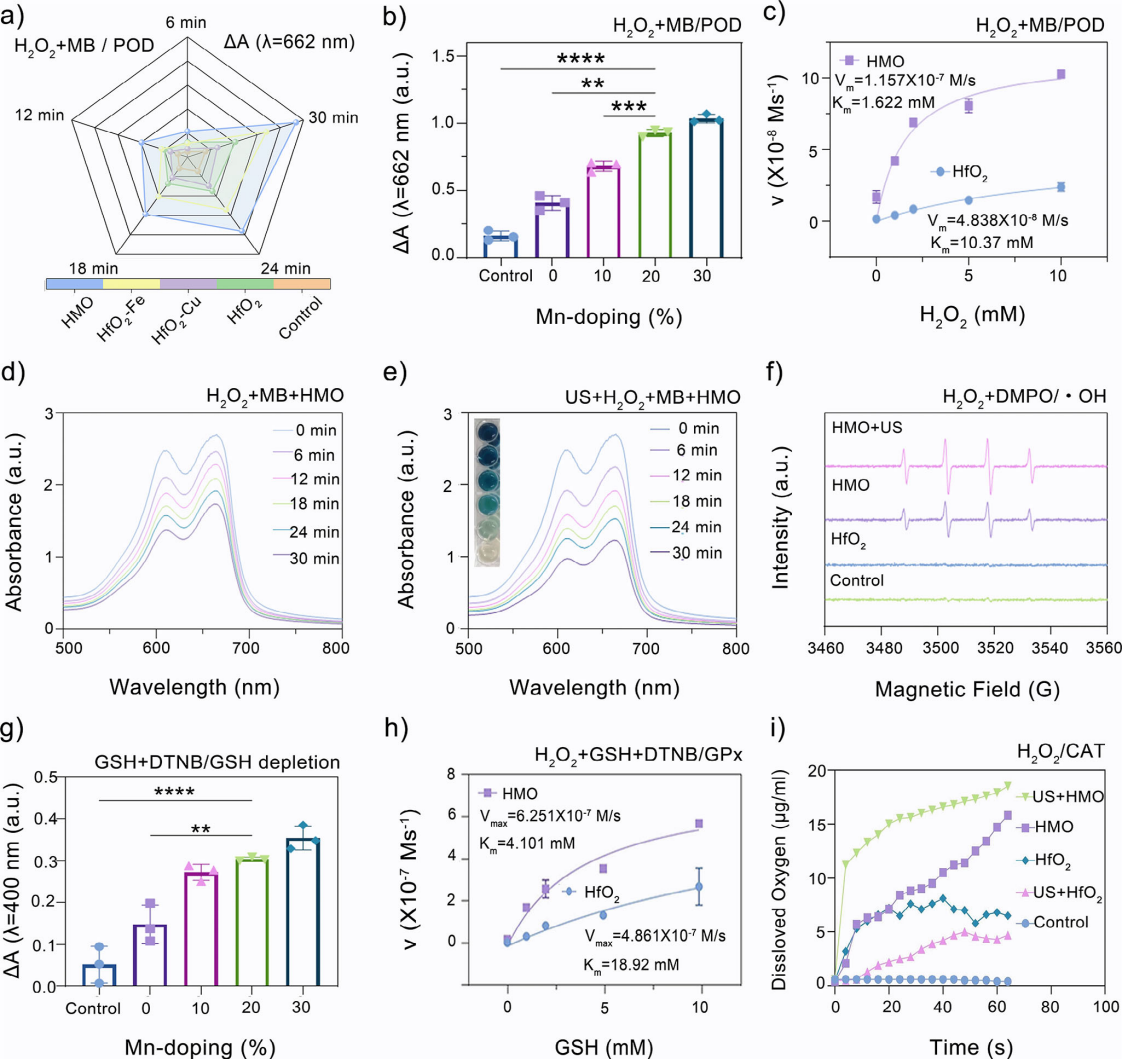
Multienzyme‐mimicking activities of HMO nanocatalysts. a) UV–vis absorbance changes of MB treated with different metal‐ion doped HfO_2_ containing H_2_O_2_ as a substrate at different times. b) UV–vis absorbance changes of MB and H_2_O_2_ exposed to HfO_2_‐Mn with different Mn‐doping contents. Data are presented as mean ± standard deviation (S.D.) (*n* = 3). c) Michaelis–Menten curves of MB treated with HMO and HfO_2_ using H_2_O_2_ as a substrate (*n* = 3). d) ·OH generation catalyzed by HMO and H_2_O_2_ using MB as a probe at different times. e) ·OH generation catalyzed by HMO and H_2_O_2_ under US irradiation (1.0 MHz, 1.0 W cm^−2^, 50% cycle) using MB as a ·OH probe at different times. f) ESR spectra of DMPO/·OH under different treatments. g) UV–vis absorbance changes of DTNB treated with GSH and HfO_2_‐Mn with different Mn‐doping contents (*n* = 3). h) Michaelis–Menten curves of DTNB and H_2_O_2_ catalyzed by HMO and HfO_2_ using GSH as a substrate (*n* = 3). i) O_2_ generation capability under different treatments using H_2_O_2_ as a substrate.

Aside from the piezoelectric catalytic effects, transition metal ions are likely to impart enzymatic enzyme‐mimicking catalytic activity to the metal‐substituted nanocatalysts. Thus, we also measured the POD‐like activity of undoped HfO_2_ and a series of doped HfO_2_ nanocatalysts, using methylene blue (MB) as an indicator with a characteristic UV–vis absorption peak at ≈662 nm. A notable tendency is that binary‐ and ternary‐doped HfO_2_ exhibited more rapid MB degradation than the single‐doped nanocatalysts regardless of the doping elements (Figure S9, Supporting Information), indicating their higher efficacy in generating hydroxyl radical (·OH) with H_2_O_2_ as the substrate. Among the binary‐doped HfO_2_, the MB degradation degree of HfO_2_ doped with Mn (such as HfO_2_‐CuMn, HfO_2_‐FeMn) is higher than that of HfO_2_ without Mn doping (HfO_2_‐CuFe). While other single‐doped HfO_2_ resulted in no significant degradation of MB, the decrease in absorbance at 662 nm was only observed in HMO (**Figure** **3**a), evidencing the critical contribution of Mn ions to the enzymatic activity of doped nanocatalysts. Subsequently, the impact of the Mn doping molar ratio was also assessed. The time‐dependent spectra revealed that the degradation percentage of MB increased with the doping content of Mn elements (Figure 3b). Despite the higher POD‐like activity of HfO_2_‐Mn_30%_, HMO with 20% Mn doping was selected for nanocatalytic applications due to their higher sonopiezoelectric ROS generation efficacy.

Furthermore, the POD‐mimicking catalytic efficacy of undoped and doped HMO was further compared by estimating their Michaelis–Menten constant (*K*_m_) and maximal initial velocity (*V*_max_). It was calculated that HMO featured a significantly lower *K*_m_ (1.622 mm) and higher *V*_max_ (1.157 × 10^−7^ M s^−1^) than doping‐free HfO_2_ (*K*_m_ = 10.37 mm, *V*_max_ = 4.838 × 10^−8^ M s^−1^) (Figure 3c). This enhanced catalytic efficiency is primarily attributed to the incorporation of Mn ions in the doped nanocatalysts, which promotes efficient effective ·OH generation within the H_2_O_2_‐rich TME. The pH‐dependent catalytic activity of HMO was further confirmed by distinct visual changes observed in the reaction products under different pH conditions (Figure S10, Supporting Information). The pronounced higher efficacy under acidic conditions makes them particularly favorable for tumor‐specific nanocatalytic therapy. Additionally, the sonopiezoelectric effects of HMO are expected to further boost their POD‐mimicking catalytic activity. Notably, the decrease in absorbance of MB treated with HMO under US irradiation was notably greater than that treated without US irradiation (Figure 3d,e), indicating sono‐enhanced catalytic degradation. ESR measurements further confirmed that the characteristic quartet ESR signals (with a 1:2:2:1 intensity ratio) of ·OH radicals were significantly more pronounced under US activation (Figure 3f). Collectively, these findings indicate that the developed HMO nanocatalysts possess synergistic sonopiezoelectric and enzyme‐mimicking catalytic capacity for efficient ROS generation, which benefits from the optimized Mn doping in HfO_2_ NPs.

Previous studies have shown that glutathione (GSH), an antioxidant that is overexpressed by tumor cells, would consume sono‐generated ROS, compromising the therapeutic efficacy of sonopiezoelectric therapy.^[^ ^51^ ^]^ To evaluate the GSH depletion capability of doped HfO_2_, we used 5,5′‐dithiobis (2‐nitrobenzoic acid) (DTNB) as a probe. The spectral analysis revealed a decrease in the absorbance intensity of reduced DTNB at ≈400 nm with increasing Mn doping levels from 0% to 30% (Figure 3g; and Figure S11, Supporting Information), indicating that the enhanced GSH depletion capacity of HMO is likely attributable to Mn doping. Moreover, the amplified GPx activity of HMO compared to bare HfO_2_ NPs was verified by the quantitative results of *K*_m_ and *V*_max_ (Figure 3h). It is well known that the overproduced H_2_O_2_ in TME could be decomposed into O_2_ by CAT‐like nanozymes.^[^ ^52^ ^]^ We then verified whether HMO possesses CAT‐mimicking activity in the presence of H_2_O_2_ by using a dissolved‐oxygen meter. Not surprisingly, a rapid oxygen evolution was detected when HMO reacted H_2_O_2_ with under ambient conditions (Figure 3i). Similarly, the oxygen production capability of doped HfO_2_ increased with the doping ratio of Mn element (Figure S12, Supporting Information). It was also noted that both GSH depletion ability and CAT‐like activity of HMO and 30% Mn‐doped HfO_2_ showed no significant difference. Taken together, HMO with optimized ROS generation and multiple enzyme‐mimicking activity was selected as the dual‐functional nanocatalyst for subsequent biological applications, in which their biomimetic oxygen generation and GSH depletion ability would augment the oxidative damage induced by sonopiezoelectric and POD‐like catalytic effects.

### 2.4 Theoretical Analysis on the Catalytic Mechanisms of HMO Nanocatalysts

**Figure 4:**
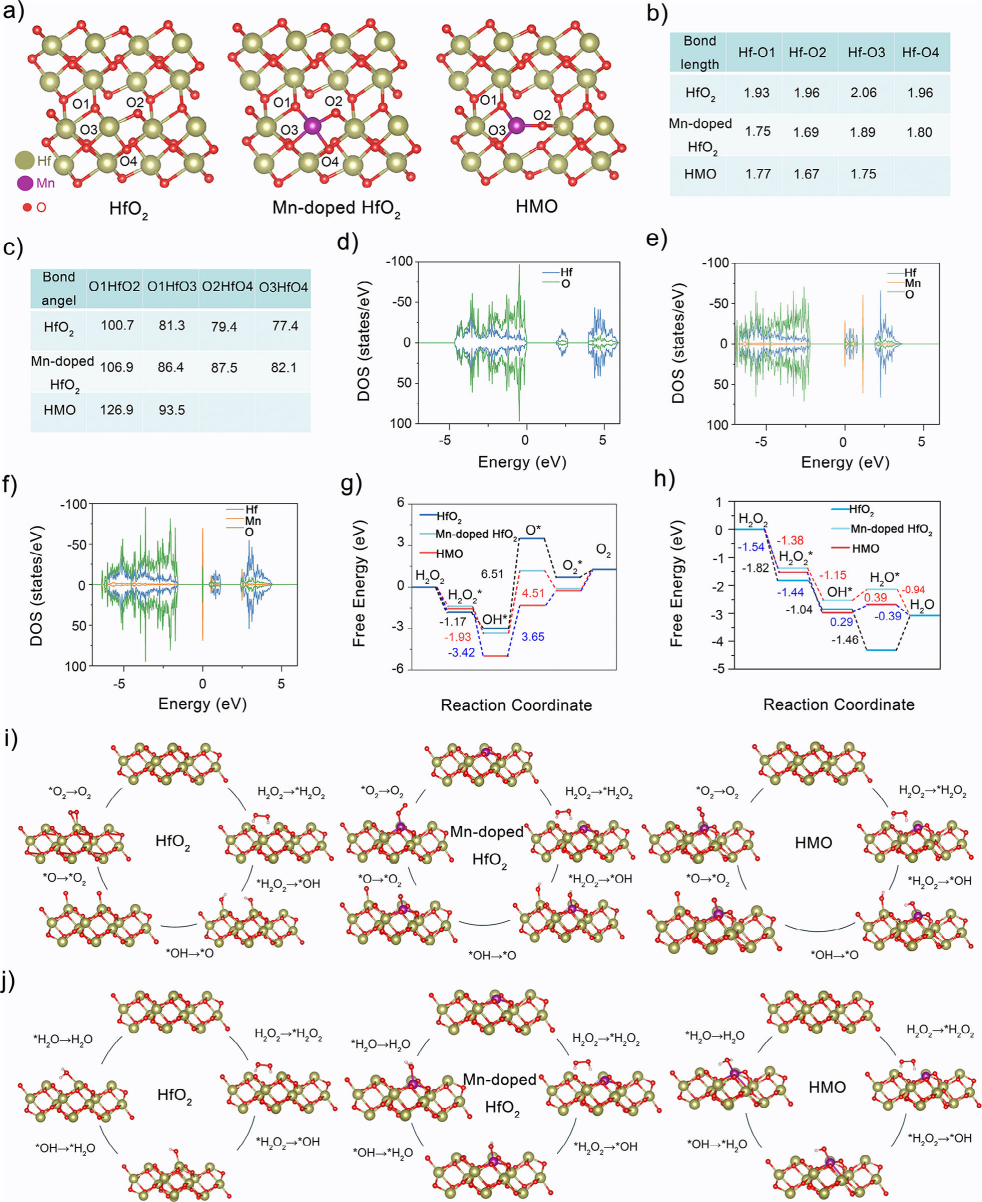
Theoretical simulations on the catalytic mechanisms of HMO nanocatalysts. a) Geometrically optimized structures of pure HfO_2_, Mn‐doped HfO_2_ (without oxygen vacancies), and HMO. b) Bond lengths and c) bond angles of HfO_2_, Mn‐doped HfO_2_, and HMO_._ Density of states (DOS) profiles of d) HfO_2_, e) Mn‐doped HfO_2_, and f) HMO. g) Energy diagrams of the catalytic pathways of H_2_O_2_ in the HfO_2_, Mn‐doped HfO_2_, and HMO. h) Energy diagrams of the Fenton catalysis pathways in the HfO_2_, Mn‐doped HfO_2_, and HMO. i) The CAT‐like catalytic pathways of modeling surface configurations in the HfO_2_, Mn‐doped HfO_2_, and HMO. j) The POD‐like catalysis pathways of modeling surface configurations in the HfO_2_, Mn‐doped HfO_2_, and HMO.

To gain deeper insight into the sonopiezoelectric and enzymatic catalytic effects of HMO, theoretical calculations were conducted. HfO_2_ can generate a piezoelectric potential due to the separation of positive and negative charge centers when subjected to an external force. Given that the enhanced catalytic efficacy of HMO hypothetically arises from Mn substitution and generated OVs, three structural models were constructed: pristine HfO_2_, Mn‐doped HfO_2_ (without OVs), and HMO, to elucidate their specific contributions. The optimization diagrams revealed significant structural deformation in HMO compared to the other two models (**Figure** **4**a). Accordingly, changes in atomic bond length and bond angle in HfO_2_ before and after Mn doping were analyzed (Figure 4b,c). The results showed that the Hf*─*O bond length decreased, while the O*─*Hf*─*O bond angle increased after replacing Hf^4+^ with Mn, relative to the pristine HfO_2_. Furthermore, these changes were more pronounced in HMO, with a further reduction in bond length and an increase in bond angle. These calculations indicate that both Mn doping and the presence of oxygen vacancies contribute to lattice distortion in HMO, consistent with previous observations from HRTEM and XRD analyses.

Given that Mn substitution and structural reconstruction are likely beneficial for the generation of oxygen vacancies on the surface of HMO, we calculated the oxygen vacancy formation energy (*E*_Ov_). It was found that the *E*_Ov_ of HMO*_v_* was calculated to be 1.7 eV. This vacancy formation energy is lower than 4.6 eV of HfO_2_, indicating that Mn doping is beneficial for producing OVs, which was also confirmed by the XPS and EPR results (Figure 1n,p). The density of states (DOS) for ideal HfO_2_, Mn‐doped HfO_2_, and HMO were also analyzed. HMO exhibited distinct defect states between the conduction band (CB) and valence band (VB) compared to ideal HfO_2_ (Figure 4d–f). As a consequence, sono‐triggered electrons are more readily excited to the CB and transferred to the surface of HMO nanocatalysts for redox reactions. At the same time, the introduced defects also trap these electrons to enhance electron–hole pair separation and suppress their recombination. These findings suggest that Mn doping induces lattice distortion and creates defect bands in HMO, thereby boosting their sonopiezoelectric catalytic efficacy.

To elucidate the enzymatic activity mechanism, key intermediate structures and free energy profiles were analyzed using density functional theory (DFT) calculations. In the CAT route, pure HfO_2_, Mn‐doped HfO_2_, and HMO all showed comparable energy changes in the initial steps, which involve H_2_O_2_ adsorption and subsequent O*─*O bond cleavage (Figure 4g,i). Notably, the subsequent deprotonation step on HMO exhibited a larger energy drop of 3.42 eV than HfO_2_ and Mn‐doped HfO_2_, implying that the decomposition by HMO with OVs is thermodynamically more favorable. Moreover, the formation of O* on the HMO surface, which is the rate‐determining step for CAT‐like catalytic reaction, overcame the lowest energy barrier (3.56 eV) compared to other tested configurations. These results corroborate that the CAT‐like activity of HMO*_v_* was significantly enhanced due to the formation of OVs. For the POD route (Figure 4h,j), HfO_2_, Mn‐doped HfO_2_, and HMO also showed negative binding energy for the surface adsorption of H_2_O_2_. Subsequently, energy barriers to produce •OH from *OH by HMO was 0.29 eV, which is significantly lower than HfO_2_ and Mn‐doped HfO_2_, These results substantiate that HMO with intrinsic OVs possess a higher POD‐like activity.

Based on theoretical calculations and experimental results, a plausible mechanism was proposed to explain the sonopiezocatalytic/enzymatic effects of HMO. Mn substitution in the crystal structure of HfO_2_ induces lattice distortions and charge imbalances, thereby increasing polarity and generating built‐in electric fields that improve charge carrier separation under the US.^[^ ^53^ ^]^ Moreover, Mn substitution generates abundant oxygen vacancies (OVs), which introduce impurity states and narrow the bandgap of doped HfO₂. These OVs act as charge traps, suppressing the recombination of sono‐generated electron–hole pairs and thereby enhancing charge separation efficiency, which significantly boosts sonopiezoelectric catalytic performance.^[^ ^54^, ^55^, ^56^ ^]^ Meanwhile, OVs improve the adsorption of intermediate substrates, accelerating enzymatic catalytic reactions. Thus, optimizing the Mn doping ratio enables HMO to implement effective dual‐mode nanocatalytic therapy.

### 2.5 In Vitro Therapeutic Efficacy of HMO Nanocatalysts

**Figure 5:**
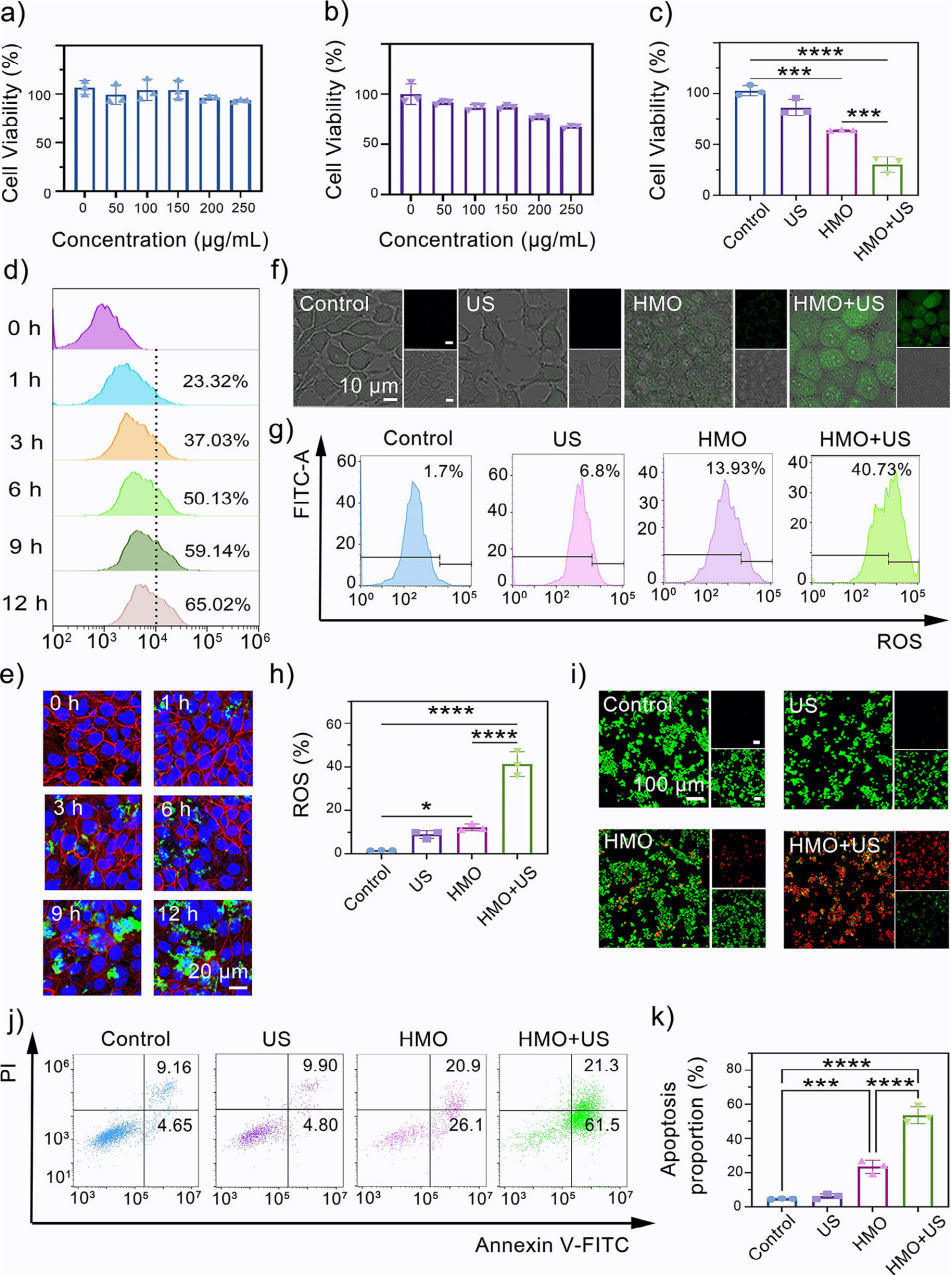
In vitro therapeutic efficacy of HMO nanocatalysts. Cell viability of a) human umbilical vein endothelial cells (HUVECs). b) 4T1 cells incubated with different HMO concentrations for 24 h, and c) 4T1 cells subjected to different treatments: I) Control; II) US; III) HMO; IV) HMO+US (*n* = 3). Data are presented as mean ± standard deviation (S.D.). d) Confocal laser scanning microscopy (CLSM) images of cellular phagocytosis behavior in 4T1 cells incubated with FITC‐labeled HMO at different time intervals. e) Flow cytometry (FCM) analysis of cellular phagocytosis behavior in 4T1 cells incubated with FITC‐labeled HMO at different time intervals. f) CLSM images of ROS generation in 4T1 cells using 2,7‐dichlorofluorescein diacetate (DCFH‐DA) as probe after different treatments. g,h) FCM analysis of ROS generation in 4T1 cells after different treatments (*n* = 3). i) CLSM images of apoptosis in 4T1 cells costained with Annexin V‐FITC/PI after different treatments. j,k) FCM analysis of apoptosis in 4T1 cells costained with Annexin V‐FITC/PI after different treatments (*n* = 3).

Considering the mutually enhanced catalytic effects of HMO, their in vitro therapeutic efficacy was evaluated next. First, we evaluated the cytotoxicity of HMO on human umbilical vein endothelial (HUVEC), mouse fibroblast (L‐929), human embryonic kidney 293 (HEK‐293), and RAW 264.7 cells using the standard CCK‐8 assay. The data revealed that HMO showed minimal cytotoxicity on these cells even at a high concentration of 250 µg mL^−1^ (**Figure** **5**a; and Figure S13, Supporting Information). Subsequently, we evaluated the anticancer effect of HMO in vitro. On the other hand, the viability of 4T1 cells slightly decreased with the increased concentration of HMO (Figure 5b), which may be explained by the enzyme‐mimicking activity that stems from Mn substitution. Additionally, the anticancer efficacy of HMO under US irradiation was also assessed. Both the control and US alone groups exhibited negligible cytotoxicity. In contrast, the HMO + US group demonstrated notable cell damage, indicating the synergistic effects of sonopiezoelectric and enzymatic activities in promoting 4T1 cell killing (Figure 5c). Next, the intracellular uptake of the nanocatalysts by cancer cells was also investigated by visualizing the localization of FITC‐labeled HMO. As seen in Figure 5d, confocal laser scanning microscope (CLSM) images demonstrated that the green fluorescence in 4T1 cells became much brighter with the prolonged incubation time. This is consistent with the results observed by the flow cytometry (FCM) analysis that the fluorescent intensity of 4T1 cells continuously increased from 1 to 12 h incubation (Figure 5e; and Figure S14, Supporting Information), suggesting the efficient internalization of HMO into cancer cells.

Subsequently, the intracellular ROS generation capacity of HMO was determined in 4T1 cells. 2,7‐dichlorofluorescein diacetate (DCFH‐DA) was selected as an indicator to distinguish the intracellular ROS generation.^[^ ^57^ ^]^ Strong green fluorescence signals were observed in cells treated with HMO and HMO upon US irradiation (Figure 5f), whereas no significant fluorescence appeared for the control or US group. The data validated that the treatment of HMO + US enabled specific ROS generation in cancer cells, as further supported by FCM analysis (Figure 5g,h). Additionally, the therapeutic efficacy of HMO was assessed using an Annexin V‐FITC/propidium iodide (PI) staining assay. The control and HMO groups showed predominant green fluorescence, indicating high 4T1 cell viability (Figure 5i). However, the US + HMO group displayed strong red fluorescence across the entire observed area, indicating severe cell damage caused by combined sonopiezoelectric and enzymatic effects. Annexin V‐FITC/PI staining followed by FCM analysis further confirmed these findings (Figure 5j,k). While no red fluorescence was observed in the control or US‐only groups, a portion of red spots were evident in the HMO and HMO + US groups, indicating cell membrane disruption and apoptosis induced by the nanocatalytic therapy.^[^ ^58^ ^]^ Therefore, these results highlight that the developed HMO nanocatalysts can be an effective nanocatalyst that combines sonopiezoelectric and enzymatic effects for tumor therapy.

### 2.6 PANoptosis Activation and Immunogenic Response by HMO Nanocatalysts

**Figure 6:**
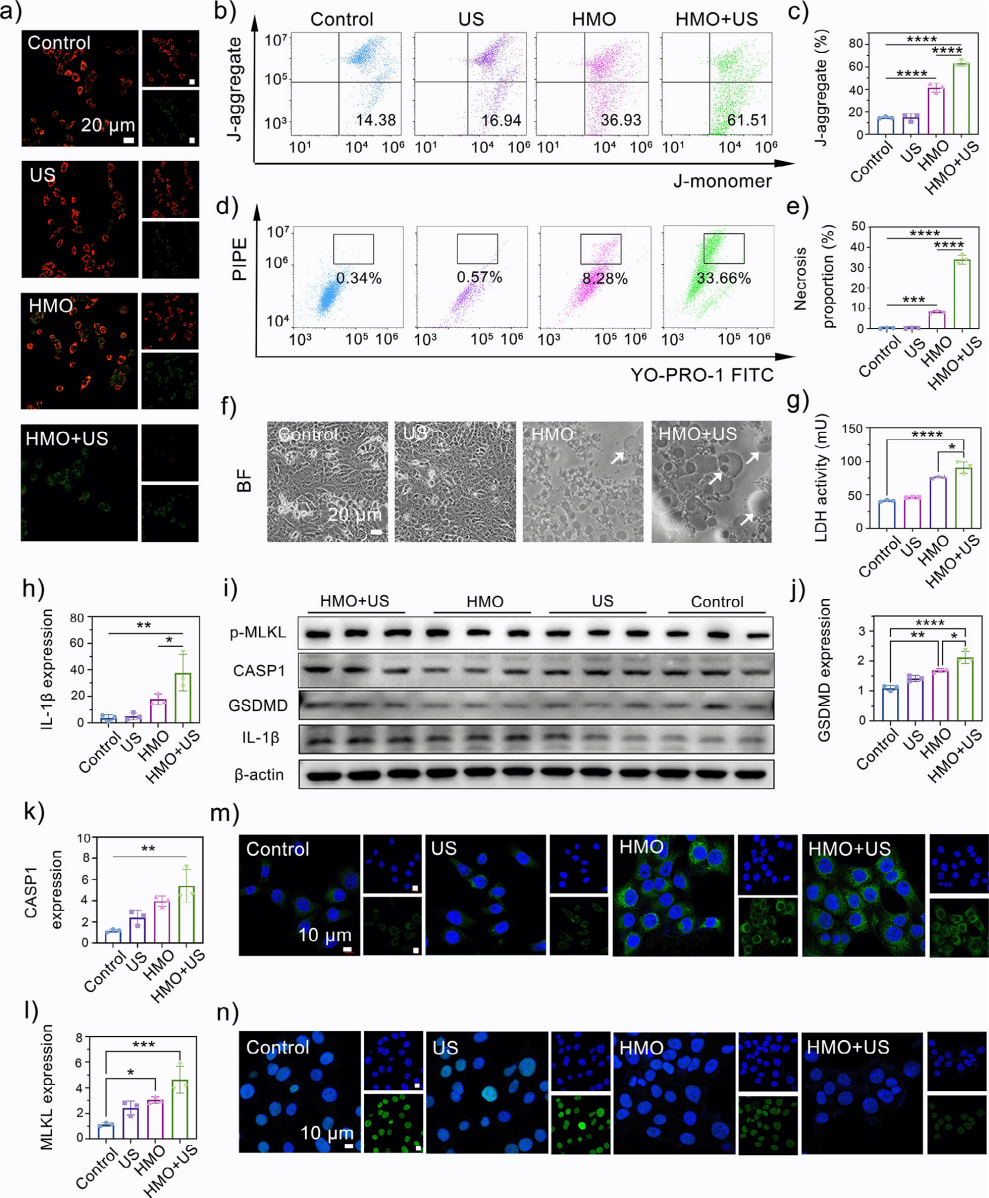
PANoptosis activation and immunogenic response by HMO nanocatalysts. a) CLSM images of mitochondrial membrane potentials (MMPs) in 4T1 cells using JC‐1 as a probe for different groups: I) Control; II) US; III) HMO; IV) HMO+US. b,c) FCM analysis of MMPs in 4T1 cells after different treatments. Data are presented as mean ± standard deviation (S.D.) (*n* = 3). d,e) FCM analysis of necrosis in 4T1 cells after different treatments (*n* = 3). f) Bright‐field microscopy image of 4T1 cells after different treatments. g) Lactate dehydrogenase (LDH) in 4T1 cells after different treatments (*n* = 3). h) Polymerase chain reaction (PCR) of IL‐1β expression after different treatments (*n* = 3). i) Western blot (WB) analysis of p‐MLKL, Caspase‐1 (CASP1), GSDMD, and IL‐1β expressions after different treatments (*n* = 3). PCR of j) GSDMD, k) CASP1, l) MLKL expression (*n* = 3). CLSM images of m) CRT and n) HMGB1 exposure of 4T1 cells after different treatments.

The therapeutic mechanisms of HMO nanocatalysts were investigated in detail. As apoptosis can cause oxidative injury to mitochondria and lead to the loss of membrane potential, JC‐1 staining was first performed to monitor mitochondrial dysfunction.^[^ ^59^ ^]^ As shown in **Figure** **6**a, the HMO group exhibited slightly weakened red fluorescence compared to the control group, suggesting a certain degree of mitochondrial damage. The HMO + US group displayed the strongest green fluorescence under US irradiation, which evidences severe deconstruction of the mitochondrial membrane after the combined treatment. FCM results further validated the same tendency of a higher green fluorescence intensity in the HMO + US group (Figure 6b,c). Moreover, the intracellular adenosine triphosphate (ATP) levels were assessed following various treatments. Notably, HMO + US treatment resulted in a notable reduction in ATP production, implying severe mitochondrial dysfunction triggered by piezocatalytic therapy (Figure S15, Supporting Information). It is well‐documented in previous publications that the oxidative damage of mitochondria is implicitly associated with cell apoptosis, necroptosis, and pyroptosis. Thus, to explore the potential of HMO upon US irradiation in activating PANoptosis, cells were collected after the treatments, stained with YO‐PRO‐1 (YP1)/PI, and analyzed by FCM. HMO with US stimulation significantly improved the proportion of dead cells (Figure 6d,e), including both YP1‐positive cells (apoptosis or necroptosis) and PI‐positive cells (necroptosis or pyroptosis).^[^ ^60^ ^]^ This observation, along with the above‐demonstrated FCM results, corroborates the induction of apoptosis, necroptosis, and pyroptosis by the combined nanocatalytic therapy.

To elucidate the pyroptosis induction effects of HMO, the cell morphology of 4T1 cells was directly visualized by bright‐field microscope imaging. No abnormal morphological alternation was observed in the control and US groups. In sharp contrast, the images revealed notable bubbles on the surface of the plasma membrane in the HMO + US group (Figure 6f), which is a hallmark feature of cell pyroptosis. As a consequence of membrane rupture, the increase of IL‐1β expression and LDH release were more pronounced in the case of HMO upon US irradiation than in other groups (Figure 6g,h). We then characterized the cell death type triggered by HMO under US stimulation using western blot (WB) and polymerase chain reaction (PCR) analyses. The combined treatment upregulated the expression of pivotal protein molecules associated with pyroptotic, apoptotic, and necroptotic pathways (Figure 6i; and Figure S16, Supporting Information). In contrast, the control and US groups showed relatively lower expression of the pyroptotic protein GSDMD (Figure 6j), apoptotic protein caspase‐1 (CASP1) (Figure 6k), and necroptotic protein MLKL (Figure 6l). Collectively, these findings suggest that HMO upon US irradiation effectively induces inflammatory PANoptotic cell death through cooperative activation of multiple PCD pathways, highlighting the advantages of combined sonopiezoelectric/enzymatic effects.^[^ ^36^ ^]^

The occurrence of PANoptosis would generally lead to immunogenic cell death (ICD) of tumor cells. We next examined the exposure of calreticulin (CRT) and the release of high mobility group protein B1 (HMGB1), which are two typical hallmark events of ICD.^[^ ^61^ ^]^ Treatment with HMO alone can induce a moderate upregulation of CRT and HMGB1 secretion in 4T1 cells compared to the control and US groups (Figure 6m,n). Moreover, markedly improved levels of CRT and HMGB1 were observed in cells treated with HMO plus US irradiation, as evidenced by the brighter green fluorescence in CLSM images. These results indicated that the synergetic catalytic effect of HMO upon US irradiation could enhance ICD to activate an antitumor immune response.

### 2.7 Therapeutic Mechanism Analyses of SonoPiezoelectric/Enzymatic Catalytic Therapy

**Figure 7:**
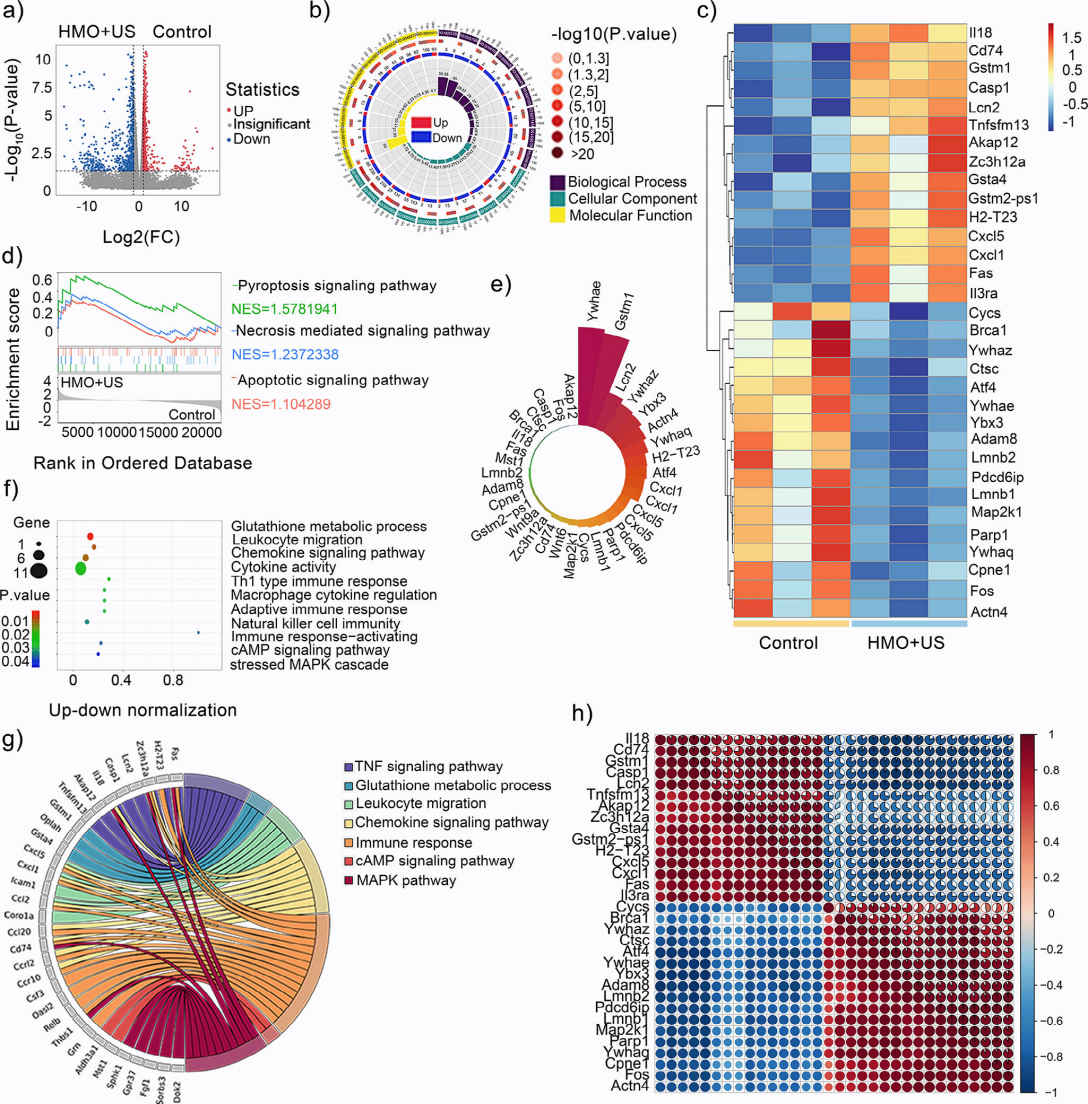
Therapeutic mechanism analyses of HMO‐mediated sonopiezoelectric/enzymatic catalytic therapy. a) Volcano plot of genes upregulated or downregulated among the differentially expressed genes in HMO + US and control groups. b) Circos plots of Gene Ontology (GO) analysis in the two groups. c) Heatmap of the significant differentially expressed proteins. d) Gene Set Enrichment Analysis (GSEA) plots of the Reactome enrichment results. e) Nightingale rose chart of the two groups. f) Bubble plot of the GO enrichment results. g) Chordal plot of Kyoto Encyclopedia of Genes and Genomes (KEGG) analysis in the two groups. h) Pearson's correlation plot of the two groups.

To further investigate the immune activation and PANoptosis‐related mechanism of HMO, RNA transcription analysis was performed on 4T1 cells subjected to US + HMO treatment and control conditions. The analysis identified 849 differentially expressed genes (DEGs) in the experimental group compared to the control group, providing insights into the molecular response to US + HMO treatment (**Figure** **7**a). The Kyoto En‐cyclopedia of Genes and Genomes (KEGG) and Gene Ontology (GO) enrichment analysis were performed to predict the molecular function, cellular component, and biological process of the DEGs (Figure 7b,g). As depicted in Figure 7d, the reactome analysis and the result in the Gene Set Enrichment Analysis (GSEA) form illustrated that differential genes were significantly enriched in signaling pathways related to pyroptosis, apoptosis, and necrosis, proving the potential of PANoptosis occurrence after nanocatalytic therapy.

Several key upregulated genes are likely associated with PANoptosis activation and immune modulation, including Cxcl1, Cxcl5, Cd74, and Gstm1. Cxcl1 and Cxcl5 are chemokines involved in promoting inflammation and immune cell recruitment. Cxcl1 enhances the differentiation of killer T cells and M1 polarization of macrophages,^[^ ^62^ ^]^ while Cxcl5 amplifies the inflammatory response by stimulating inflammatory cells to release proinflammatory cytokines and chemokines, contributing to the regulation of immune responses.^[^ ^63^ ^]^ Cd74, a key immune response marker, plays a pivotal role in antigen presentation and is clinically relevant for monitoring immune activation.^[^ ^64^ ^]^ Gstm1 is a critical enzyme involved in oxidative stress regulation, playing a key role in GSH biosynthesis and lipid peroxide detoxification for inhibiting ferroptosis,^[^ ^65^, ^66^, ^67^ ^]^ which was significantly upregulated in the US + HMO treatment. In addition, the over‐expression of Lcn2 and Akap12, which are closely associated with the induction of ferroptosis and regulation of tumor autophagy, was observed in the HMO + US group. These findings further confirmed the potential roles of HMO upon US irradiation in participating in other PCD pathways (Figure 7c).^[^ ^68^, ^69^, ^70^ ^]^

To explore the induction effects of HMO on ferroptosis upon US irradiation, the GSH levels and ROS generation capabilities were evaluated. Bare HfO_2_ and classic piezocatalysts BaTiO_3_ were employed as control groups.^[^ ^71^ ^]^ After the treatments, HMO + US demonstrated a significant elevation in terms of GSH assumption and ROS generation compared to other groups (Figures S17 and S18, Supporting information), which might be explained by their combined sonopiezoelectric/enzymatic catalytic effects. Moreover, we then evaluated the expression of uppermost protein molecules relevant to ferroptosis by WB. The downregulated expression of glutathione peroxidase 4 (GPX4) was observed in the HMO + US group, suggesting that the combined nanocatalytic therapy holds the potential to induce ferroptosis of tumor cell. However, microtubule‐associated protein 1 light chain 3 (LC3) expression, which is a distinct characteristic of autophagy,^[^ ^72^ ^]^ exhibited no significant difference between various treatments, implying that HMO upon US irradiation may be incapable of triggering autophagic cell death (Figure S19, Supporting Information).

The gene abundance map further demonstrated significant activation of the immune‐related pathways in the HMO + US group (Figure 7e,h), including cytokine activity and chemokine signaling, suggesting a robust in vivo immune response. The results showed a considerable enrichment of immune‐related pathways (Figure 7f), such as the TNF signaling pathway, cAMP signaling pathway, cytokine activity, Th1 type immune response, chemokine signaling pathway, and other immune‐related pathways, after the HMO + US treatment. Protein–protein interaction network interactions also revealed the expression of key genes associated with immune infiltration (Figure 7g).^[^ ^73^ ^]^ The upregulation of several immune‐related genes, including Ccl2 and Cd74, highlighted a marked increase in immune infiltration, particularly in dendritic cell (DC) maturation, M1 macrophage polarization, and T‐cell differentiation.^[^ ^74^ ^]^ The upregulation of IL‐18, an analogue of IL‐1β, in the treatment group, may be induced by interferon, which can induce T cells to produce high levels of IFN‐γ, suggesting its involvement in immune system response.^[^ ^75^ ^]^ Our findings demonstrate that US + HMO treatment activates PANoptosis and amplifies the immunoactivation efficacy by upregulating key PANoptosis‐related genes and promoting immune infiltration.

### 2.8 HMO‐Mediated SonoPiezoelectric/Enzymatic Catalytic Therapy Inhibits Tumor Growth

**Figure 8:**
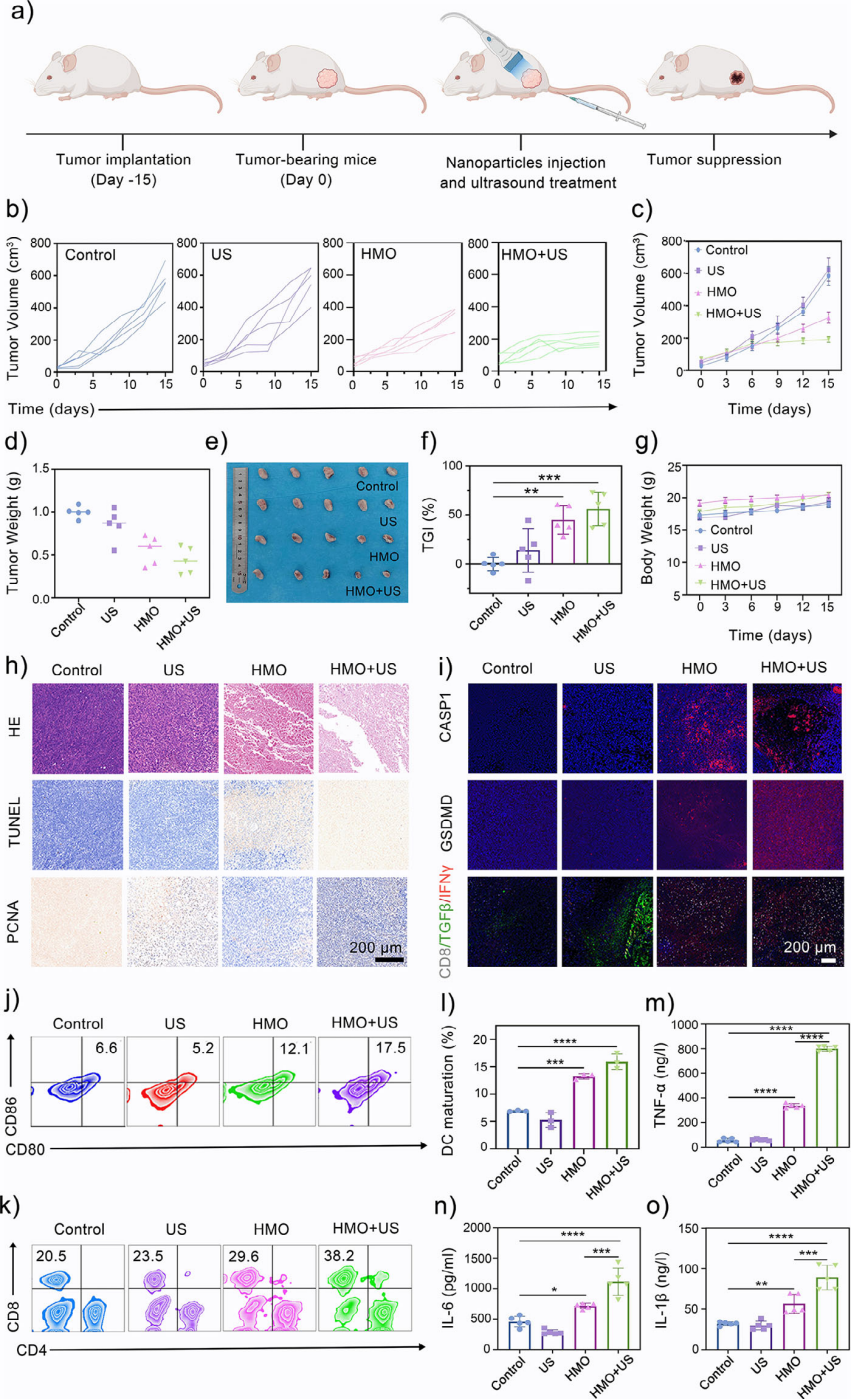
HMO‐mediated sonopiezoelectric/enzymatic catalytic therapy inhibits tumor growth. a) Schematic illustration for the establishment and the treatment procedure for 4T1 tumor‐bearing mice model. b) Individual tumor volume curves in different groups: I) Control; II) US; III) HMO; IV) HMO + US (*n* = 5). c) Average volume change curves after different treatments. Data are presented as mean ± standard deviation (S.D.) (*n* = 5). d) Average tumor weight change curves of mice after different treatments (*n* = 5). e) Excised tumors of each group on the 15th day after different treatments (*n* = 5). f) The tumor growth inhibition (TGI) of each group on the 15th day after different treatments (*n* = 5). g) Body weights of the mice after different treatments (*n* = 5). h) H&E, TUNEL, and PCNA staining images of tumors after different treatments. i) Immunofluorescence staining of CASP1, GSDMD, and CD8/TGFβ/IFNγ of the tumor tissues after different treatments. j,l) Dendritic cells (DCs) maturation in lymph nodes quantified analyzed by FCM (*n* = 3). k) CD4^+^ and CD8^+^ T cells in tumors quantified by FCM. m–o) ELISA measures TNF‐α, IL‐6 and IL‐1β Interleukinsecretion levels after different treatments (*n* = 5).

Encouraged by the therapeutic outcome in vitro, the antitumor effect of HMO under US irradiation was further investigated in tumor‐bearing BALB/c mice. The biosafety of HMO was evaluated in terms of histological examination and hemocompatibility. The major organs (heart, liver, spleen, lung, and kidney) were extracted from mice injected with HMO at a high dosage of 25 mg kg^−1^. As shown in Figures S20 and S21, Supporting Information, no obvious pathological abnormalities were observed, demonstrating the negligible damage of HMO against normal organs. Hematological and blood biochemical analysis results revealed no significant abnormal indexes after the administration of HMO (Figure S22, Supporting Information), further suggesting the favorable biocompatibility of HMO. For the in vivo antitumor studies, 4T1‐tumor‐bearing BALB/c mice with different treatments were randomly divided into four groups (*n* = 5): 1) saline, 2) US, (3) HMO (dosage, 15 mg kg^−1^), and 4) HMO + US (dosage, 15 mg kg^−1^) (**Figure** **8**a). For the US and HMO + US groups, the tumor location was exposed to the US (1.0 MHz, 1.0 W cm^−2^, 50% cycle) for 5 min at 12 h postinjection. The body weights and tumor volumes of the mice were monitored every two days throughout the 15 days treatment period. The tumors in the saline and US treatment groups showed rapid growth (Figure 8b–e), suggesting that US irradiation had a negligible influence on tumor inhibition. Compared to the moderate tumor inhibition rate (44.4%) in the HMO group (Figure 8f), the tumor growth was drastically suppressed in the HMO + US group, with the highest inhibition rate (72.7%). The superior therapeutic outcome of HMO is possibly due to the GSH‐depleted SDT and US‐enhanced PANoptosis in tumors by HMO. In addition, no obvious weight loss was observed for the control or treatment groups (Figure 8g), suggesting minimal adverse effects of HMO nanocatalysts. To verify the antitumor effect of HMO at the histological level, a tumor specimen was randomly selected from each group for hematoxylin and eosin (H&E) and terminal deoxynucleotidyl transferase‐mediated deoxyuridine triphosphate nick end labeling (TUNEL) staining. As shown in Figure 8h, tumors treated with HMO + US exhibited the most pronounced wrinkling and a marked reduction in nuclei, indicating severe damage and widespread cell death. Furthermore, proliferating cell nuclear antigen (PCNA) immunohistochemical staining revealed significantly decreased proliferation in the HMO + US group, highlighting its strong inhibitory effect on tumor aggressiveness (Figure 8h). To further understand the therapeutic mechanisms, the expression levels of GSDMD and CASP1 were examined by immunohistochemical staining. The combined nanocatalytic treatment also showed the highest GSDMD and CASP1 expressions (Figure 8i), indicating the occurrence of PANoptosis in tumors.

Furthermore, we further evaluated the capacity of HMO to induce PANoptosis and reverse immunosuppressive TME for enhanced antitumor immunotherapy of HMO (Figure 8j,k). Since the maturation of DCs in vivo can strongly stimulate the activation and proliferation of T cells, FCM was first employed to quantify the maturity of DCs in the lymph.^[^ ^50^ ^]^ As expected, the highest maturity of the proportion of mature DCs in the lymph nodes appeared in the HMO + US group (Figure 8j,l), revealing robust antigen presentation ability and immune system activation. Similarly, HMO + US treatment increased the infiltration rate of cytotoxic T lymphocytes (CTLs) from 20.5% to 38.2% (Figure 8k). Compared to the control and US alone groups, we observed relatively high expression of TGFβ, low expression of IFN‐γ and CD8^+^ in the HMO and HMO + US groups (Figure 8i), indicative of the amplified immune response and improved infiltration of CD8^+^ T cells.^[^ ^76^, ^77^ ^]^ These findings suggested that the piezoelectric/enzymatic nanocatalytic therapy mediated by HMO upon US irradiation can exert immune‐stimulating effects to potentiate antitumor efficacy. Proinflammatory cytokines play a significant role in stimulating, recruiting, and expanding immune cells, thereby augmenting the body's immune response against tumors.^[^ ^78^ ^]^ HMO + US treatment also enhanced the secretion of antitumor cytokines, such as tumor necrosis factor‐α (TNF‐α), Interleukin (IL)‐6, and IL‐1β (Figure 8m–o). Overall, HMO effectively activated antitumor immune responses under US irradiation through the dual action of adaptive immunity triggered by PANoptosis and innate immunity, guaranteeing satisfactory inhibition effects on tumor growth.

## 3 Conclusion

In summary, HMO nanocatalysts were developed as a sonopiezoelectric nanocatalyst with multienzymatic activities for PANoptosis‐augmented sono‐immunotherapy. Transition metal engineering was proven to be an effective strategy that not only can modulate the sonopiezoelectric response of HfO_2_ nanoparticles but also render the doped nanocatalysts with multienzymatic activities that mimic POD, CAT, and GPx. Notably, 20% Mn substitution in HfO_2_ resulted in oxygen vacancies and lattice distortions, achieving optimal dual‐functional catalytic efficacy. These features enabled HMO to generate ROS efficiently under US irradiation, relieve hypoxia, and deplete GSH within the tumor microenvironment, amplifying oxidative stress and inducing cancer cell death. In addition, HMO nanocatalysts demonstrated excellent biocompatibility and effectively triggered PANoptosis, including apoptosis, necroptosis, and pyroptosis, upon US irradiation. ICD markers, such as CRT and HMGB1, were significantly upregulated, promoting immune system activation. RNA sequencing and pathway enrichment analysis revealed that HMO modulated immune‐related pathways and oxidative stress responses. In vivo studies confirmed the targeted accumulation of HMO at tumor sites, where its combination with US irradiation effectively inhibited tumor growth and activated robust antitumor immunity. This study highlights HMO as a promising nanocatalyst that synergistically combines sonopiezoelectric and enzyme‐mimicking catalytic activities to induce PANoptosis, providing an effective sono‐triggered immune activation strategy for cancer treatment. Despite the enormous potential of HMO, it should be noted that the clinical translation of HMO still faces challenges in batch‐to‐batch consistency and long‐term safety, which necessitates the development of high‐throughput synthetic technology and meticulous biological evaluations.

## Conflict of Interest

The authors declare no conflict of interest.

## Supporting information



Supporting Information

## Data Availability

The data that support the findings of this study are available from the corresponding author upon reasonable request.
