# Supplementary material for: Dopant‐Regulated Piezocatalysts Evoke Sonopiezoelectric and Enzymatic PANoptosis for Synergistic Cancer Therapy
Source: Adv Sci (Weinh). 2025 Mar 8;12(17):2500406. doi: 10.1002/advs.202500406 (PMC12061309; doi:10.1002/advs.202500406)
Supplement: Supplementary file 1 — Supporting Information [file ADVS-12-2500406-s001.docx]

Supporting Information

**Dopant-Regulated Piezocatalysts Evoke Sonopiezoelectric and Enzymatic PANoptosis for Synergistic Cancer Therapy**

*Linhong Zhong*^#^*, Xun Guo*^#^*, Liming Deng, Xiaoting Wang, Hongye He, Nianhong Wu, Rui Tang, Liang Chen*********, Yu Chen*********,* *Pan Li********

^#^ These authors contributed equally to this work.

* Corresponding Author: Yu Chen, Pan Li, Liang Chen

**This file includes:**

Experimental sections

Figs. S1 to S22

Tables S1 to S2

**Experimental Sections**

*Chemicals*

All chemicals were used without purification because of the analytical grade of the reagents. Hafnium chloride (HfCl_4_), Sodium hydroxide (NaOH), Polyvinylpyrrolidone (PVP, K29-32), Manganese chloride tetrahydrate (MnCl_2_·4H_2_O), Iron chloride hexahydrate (FeCl_3_·6H_2_O), Copper chloride dihydrate (CuCl_2_·2H_2_O), Glutathione, N,N-dimethylformamide (DMF) were obtained from Shanghai Macklin Biochemical Technology Co., Ltd. 1,3-Diphenylisobenzofuran (DPBF), 5,5'-dithiobis-2-nitrobenzoic acid (DTNB), Methylene blue (MB) were purchased from Shanghai Sigma-Aldrich Corporation. The CCK-8 assay kit, LDH Assay Kit, 2,7-dichlorofluorescein diacetate (DCFHDA), 5,5,6,6′-tetrachloro-1,1′,3,3′ tetraethylbenzimi-dazoylcarbocyanine iodide (JC-1) staining kit, apoptosis and necrosis detection kit with YO-PRO-1 and PI, and annexin V-FITC/PI apoptosis detection kit were purchased from Beyotime Inst. Biotech. Fetal bovine serum (FBS), trypsin, Roswell park memorial institute 1640 medium, phosphate buffered saline (PBS), Human umbilical vein endothelial cells (HUVECs) and 4T1 cells were obtained from Wuhan Pricella Biotechnology Co., Ltd.

*Instruments*

Various techniques were employed for characterization. TEM and HRTEM images were performed by a JEOL F200 thermal field emission transmission electron microscope (200 kV) (JEOL, Japan). XPS analysis was obtained by a Thermo SCIENTIFIC K-Alpha instrument (Thermo Scientific, USA). XRD patterns were analyzed by a Bruker D8 Advance (Bruker, GER). UV-vis-NIR absorption spectra were obtained with a UV-3600 Shimadzu UV-vis-NIR spectrophotometer. CLSM images were captured by an FV3000 instrument (Olympus, Japan). ESR characterization was recorded with a Bruker EMXPlus-10 instrument (Bruker, GER). All experiments involving US treatment utilized a US transducer from WELLD Co., China.

*Synthesis of HMO nanocatalysts*

HMO were synthesized by a hydrothermal method. Typically, 0.03 mol/L HfCl_4_ and 0.02 mol/L MnCl_2_ were stirred at 80 °C. Subsequently, 0.1 mol/L of sodium hydroxide solution was slowly added and stirred at room temperature. Then, the mixture solution was at 180 °C for 24 h. After heated procedure, the resultant solution in Teﬂon autoclave was centrifuged and washed with deionized water for several times, and the colloidal precipitates were collected for further modiﬁcation with PVP.

*Synthesis of different metal-ions doped HfO_2_ nanocatalysts*

The diverse doped HfO_2_ nanocatalysts were synthesized by the same method as HMO. For HfO_2_, 0.05 mol/L HfCl_4_ was used as precursor. For single metal-ion doped HfO_2_, 0.03 mol/L HfCl_4_ and 0.02 mol/L FeCl_3_ or CuCl_2_ were used as precursors. For binary metal-ion doped HfO_2_, 0.03 mol/L HfCl_4_, 0.01 mol/L FeCl_3_, and 0.01 mol/L CuCl_2_ (HfO_2_-CuFe), or 0.01 mol/L MnCl_2_ and 0.01 mol/L CuCl_2_ (HfO_2_-CuMn), or 0.01 mol/L MnCl_2_ and 0.01 mol/L CuCl_2_ (HfO_2_-FeMn) were used as precursors. For ternary metal-ion doped HfO_2_, 0.03 mol/L HfCl_4_, 0.01 mol/L FeCl_3_, 0.01 mol/L CuCl_2_, and 0.01 mol/L MnCl_2_ (HfO_2_-CuFeMn) were used as precursors. For different Mn-doped HfO_2_, 0.04 mol/L HfCl_4_ and 0.01 mol/L MnCl_2_ (HfO_2_-Mn_10%_), or 0.02 mol/L HfCl_4_ and 0.03 mol/L MnCl_2_ (HfO_2_-Mn_30%_) were used as precursors, respectively. The subsequent synthesis process is identical as HMO.

*Detection of ROS Generation in Vitro*

The ^1^O_2_ generation was detected using DPBF as a probe. Briefly, 200 μg/mL of HMO solution containing DPBF (working concentration: 0.1 mg/mL) was exposed to US (1.0 MHz, 1.0 W/cm^2^, 50% cycle), and obtained the absorption intensity from a UV-vis spectrophotometer at given time points. ESR measurements were also performed to identify the type of ROS generated by HMO under US irradiation. For the generation of ^1^O_2_, HMO solution (5 mL, 200 μg/mL) containing TEMP (50 mM) was irradiated with US (1.0 MHz, 1.0 W/cm^2^, 50% cycle) for 5 min in the dark, and record the signals. For ·OH generation, HMO solution (5 mL, 200 μg/mL) containing DMPO (100 mM) by H_2_O_2_ (10 mM) as a substrate was exposed to US (1.0 MHz, 1.0 W/cm^2^, 50% cycle) for 5 min, and record the signals.

*Enzyme-Mimicking Activity of HMO*

MB was used as an indicator to assess the POD-like activity of HMO or doped HfO_2_. 200 μg/mL HMO or HfO_2_ was dispersed in different pH PBS solutions (pH 7.4, 6.5, 5.5, and 4.5) containing MB (1.5 mg/mL) using H_2_O_2_ (10 mM) as a substrate. At given time points, the UV-vis absorbance of the solution was obtained. CAT-like activity of HMO was carried out by determining the amount of dissolved oxygen. The oxygen electrode of the dissolved oxygen meter JPBJ-609 L (Leici, China) was used to assess the CAT-like activity of HMO or HfO_2_. 200 μg/mL HMO or HfO_2_ was dispersed with H_2_O_2_ (10 mM), and the amount of dissolved oxygen was recorded at given time points. And US irradiation (1.0 W/cm², 1 MHz, 50% duty cycle) was performed at given time point. DTNB was used as an indicator to assess the GPx-like activity of HMO or HfO_2_. 200 μg/mL HMO or HfO_2_ was added to DTNB (0.1 mM) and H_2_O_2_ (10 mM), where GSH (10 mM) acted as a substrate. At given time points, the UV-vis absorbance of the solution was obtained.

*GSH Depletion*

HMO (500 μg/mL) were dispersed in GSH (10 mM) solution and subsequently irradiated with US for different times. After the addition of DTNB (0.1 mM), the intensity of the absorption peak at around 400 nm was recorded at given time points by a UV-vis spectrophotometer.

*Electrochemical Tests*

Electrochemical tests were carried out on an electrochemical workstation (CHI660D) in Na_2_SO_4_ solution (0.2 M). Sample-coated ITO glass, the Ag/AgCl standard electrode, and the Pt wire served as working electrodes, counter electrodes, and reference electrodes, respectively. The Eg of HfO_2_ and HMO obtained by the Mott-Schottky tests were estimated as -0.48 and -0.2 eV respectively.

*The conduction band and valence band Calculation*

The ﬂat band potentials (E_fb_) of HfO_2_ and HMO obtained by the Mott-Schottky tests were estimated as -0.48 and -0.2 V, respectively. In n-type semiconductors, the E_fb_ values were ~0.2 V higher than the VB edges. Therefore, the conduction band (CB) edges of HfO_2_ and HMO were determine as -0.68 and -0.4 eV, respectively. The valence band (VB) edges were calculated as:

E_VB_ = E_CB_ + E_g_

where the bandgap energy of HfO_2_ and HMO derived from the Tauc plots were analyzed as 2.89 and 1.89 eV, respectively. Thus, EVB = -2.56 and -1.28 eV for HfO_2_ and HMO, respectively.

*Density Functional Theory (DFT) Calculation*

Density functional theory as implemented in the Vienna Ab-initio Simulation Package (VASP) was employed to optimize geometry structures. The exchange-correlation interactions were described by the generalized gradient approximation (GGA) in the form of the Perdew-Burke-Ernzerhof functional (PBE). A cut-off energy of 500 eV for plain-wave basis sets was adopted and the convergence threshold was 10^-4^ eV, and 0.03 eV/Å for energy and force, respectively. The van der Waals interactions were considered by the method of the Grimme (DFT+D3). The vacuum space was set to be more than 15 Å, which was enough to avoid the interaction between periodical images. The brillouin-zone integration was sampled with a Monkhorst-Pack mesh of 2 × 2 × 1 in the structural relaxation calculations. The density of states was calculated with a more accurate k-points mesh of 4 × 4 × 1.

The free energy of a gas phase molecule or an adsorbate on the surface was calculated by the equation:

G = ΔEDFT + ΔEZPE −ΔTS

Where ΔEDFT represents the total energy calculated from DFT, ΔEZPE is the vibrationale zero-point energy. T is the temperature in kelvin (298.15 K is set here), and ΔS is the entropy. ΔEZPE and ΔS of adsorbates were calculated based on vibrational frequencies calculations by using VASPKIT.

*Cellular Uptake of HMO*

Cellular uptake behaviors of HMO were evaluated using CLSM. 4T1 cells were seeded into glass bottom cell culture dishes (10^4^ cells per well) and cultured overnight. Then the cells were incubated with HMO for 1 h, 3 h, 6 h, 9 h and 12 h. After that, the cells were fixed with 4% paraformaldehyde and imaged on a CLSM. For FCM analysis, and cells were seeded in 12-well plate (10^5^ cells per well). After being incubated with HMO for 1 h, 3 h, 6 h, 9 h and 12 h, cells were harvested and resuspended in PBS for FCM analysis.

*In Vitro Cytotoxicity*

The cytotoxicity was carried out on 4T1 and HUVEC cells by MTT assay, respectively. 4T1 or HUVEC cells were seeded in a 96-well plate to adhere overnight. Next, 100 μL of HMO solution with different concentrations (0, 50, 100, 150, 200 and 250 μg/mL) was added into per well and further incubated for 24 h. Then, 100 μL of MTT solution (1 mg/mL) was added, and the cell viability was determined by a microplate reader. Additionally, 4T1 cells were divided into different groups: I) Control; II) US; III) HMO; IV) HMO+US. US exposure was employed at 1.0 W/cm² with a 50% duty cycle for 3 min. After the respective treatments, the cells were incubated for 24 h, and cell viability was treated using a CCK-8 assay kit.

*Evaluation of Intracellular ROS*

To evaluate the intracellular ROS levels, DCFH-DA was used as the probe. 4T1 cells were seeded in glass bottom cell culture dishes (10^4^ cells per well) and cultured overnight. After that, cells were divided into control, US, HMO, and HMO + US groups (n=3). For US and HMO+US groups, the cells were exposed to US (1.0 MHz, 1.0 W/cm^2^, 50% cycle, 3 min). At last, DCFH-DA probe were then added to assess intracellular ROS levels. The images within the cells was observed by CLSM. For FCM analysis, 4T1 cells (10^3^ cells per well) were cultivated into a 12-well dish for 12 h to adhere. After being treated with HMO and US, cells were harvested and resuspended in PBS for FCM analysis.

*Assessment of Mitochondria Integrity*

To evaluate the integrity of mitochondria, 4T1 cells (10^4^ cells per well) were seeded in glass bottom cell culture dishes and cultured overnight. After that, cells were divided into control, US, HMO, and HMO + US groups (n=3). For US and HMO + US groups, the cells were exposed to US (1.0 MHz, 1.0 W/cm^2^, 50% cycle, 2.5 min). At last, the cells were incubated with the JC-1 for 30 min and imaged on a CLSM. For FCM analysis, 4T1 cells (10^5^ cells per well) were cultivated into a 12-well dish for 12 h to adhere. After being treated with HMO and US, cells were harvested and resuspended in PBS for FCM analysis.

*LDH Activity Detection*

To evaluate the LDH levels, 4T1 cells (10^5^ cells per well) were cultivated into a 12-well dish overnight to adhere. Next, 4T1 cells were divided into control, US, HMO (200 μg/mL), and HMO + US (200 μg/mL) groups (n=3). For US and HMO+US groups, the cells were exposed to US (1.0 MHz, 1.0 W/cm^2^, 50% cycle, 3 min). LDH levels were detected by corresponding commercial assay kits using cell supernatants.

*Evaluation of CRT and HMGB1*

4T1 cells (10^4^ cells per well) were cultivated into a glass bottom cell culture dishes and cultured for 12 h. Next, 4T1 cells were divided into control, US, HMO (200 μg/mL), and HMO + US (200 μg/mL) groups (n=3). For US and HMO+US groups, the cells were exposed to US (1.0 MHz, 1.0 W/cm^2^, 50% cycle, 3 min). After being treated with HMO and US, the cells were incubated with CRT antibody for 2 h and then incubated with an Alexa Fluor 488 antibody for another 30 min at 37 °C. To detect the release of HMGB1, the cell membranes were disrupted using a 1% Triton/PBS solution. Additionally, HMGB1 antibody was incubated using the same method as above. At last, cells were imaged on a CLSM.

*Assessment of In Vitro Antitumor Performance*

For FCM analysis, 4T1 cells (10^5^ cells per well) were cultivated into a 12-well dish overnight to adhere. Next, 4T1 cells were divided into control, US, HMO (200 μg/mL), and HMO + US (200 μg/mL) groups (n=3). For US and HMO+US groups, the cells were exposed to US (1.0 MHz, 1.0 W/cm^2^, 50% cycle, 3 min). At last, the treated cells were treated with an annexin V-FITC/PI apoptosis detection kit and necrosis detection kit with YO-PRO-1 according to the manufacturer’s instruction. For CLSM, 4T1 cells (10^4^ cells per well) were cultivated into a glass bottom cell culture dishes and cultured for 12 h. After being treated with HMO and US, cells were imaged on a CLSM.

*Quantitative PCR for Gene Expression Analysis*

4T1 cells were seeded in 6-well plates (10^6^ cells per well), and cultivated overnight. Then cells were divided into control, US, HMO (200 μg/mL), and HMO + US (200 μg/mL) groups (n=3). For US and HMO + US groups, the cells were exposed to US (1.0 MHz, 1.0 W/cm^2^, 50% cycle, 3 min). After that, the cells were collected and the Caspase1, GSDMD, IL-1β and MLKL transcription levels were analyzed by qPCR.

*Western Blotting (WB)*

4T1 cells were seeded in 6-well plates (10^6^ cells per well), and cultured for 12 h. Then cells were exposed to different treatments. After that, cells were washed and used RIPA buffer containing protease inhibitors to extract protein. 30 μg proteins of each group were loaded and run through sodium dodecyl sulfate-polyacrylamide gel electrophoresis. Proteins were transferred to the PVDF membrane and then blocked in TBS-T solution containing 5% BSA for 1 h. Thereafter, the membrane was incubated with appropriate primary antibody overnight at 4 °C. After incubation with secondary antibodies conjugated to horseradish peroxidase (HRP), protein signals were detected by enhanced chemiluminescence reagent. GAPDH and ACTIN were employed as protein loading control. To test the PANoptosis related signaling pathway activation, measuring the phosphorylation levels of Caspase1, GSDMD, IL-1β, and MLKL using WB.

*mRNA Sequencing and Analysis*

4T1 cells were seeded in 6-well plates (10^6^ cells per well), and cultured. After the treatments, RNA was extracted from the samples using TRIzol reagent (Invitrogen). Each experiment was conducted in triplicate, and RNA sequencing was performed by LC-Bio Technologies (Hangzhou, China).

*Assessment of In Vivo Biocompatibility*

Healthy female BALB/c nude mice (4-6 weeks) were randomly divided into five groups (n=5). The mice were intravenously injected with saline or HMO (25 mg/kg), blood samples were collected at given time points (0, 3, 7, 14, and 28 days) for analysis of hematological parameters and liver and kidney functions. Besides, the mice were euthanized, and the main organs (liver, spleen, lung, heart, and kidney) were collected for H&E staining at given time points (0, 3, 7, 14, and 28 days).

*In Vivo Anticancer Performance*

To establish a 4T1 breast cancer model, healthy female BALB/c nude mice (4-6 weeks) were subcutaneously injected with 4T1 breast cancer cells. When the tumor volume reached 50 mm^3^, the mice were divided into four groups (n=5) as follows: 1) saline, 2) US, 3) HMO, and 4) HMO + US. The mice were administered intravenously with saline or HMO solution. For in vivo US treatment, the mice were intravenously injected with HMO (dosage: 15 mg/kg) on day 0, 1, 3, 5 and 7, and US irradiation (1.0 MHz, 1.0 W/cm^2^, 50% cycle, 5 min) was intermittently performed at 24 h post-injection. The tumor volume and body weight of mice were measured every two days (volume (V) = width^2^ × length/2). The plasma was isolated from mice serum through retinal orbital bleeding for evaluating circulating plasma cytokine levels. IL-1β, TNF-α, and IL-6 were quantified using ELISA kit. The mice were euthanized, and the tumors were extracted and weighed after 14 days of treatment. Besides, the tumors and main organs (liver, spleen, lung, heart, and kidney) were collected for H&E, TUNEL, immunohistochemical, and immunofluorescence staining (for Caspase1, GSDMD, IL-1β, PCNA). Furthermore, to evaluate the maturation of DC cells, lymphocytes were harvested and stained with anti-CD45, CD11C, CD80 and CD86 antibody for the percentages of CD80^+^ CD86^+^ DCs by FCM analysis. Additionally, the tumors were also excised for CD4^+^ and CD8^+^ T cell staining.

*Statistical Analysis*

Quantitative data were presented as mean ± standard deviation (mean ± SD). Means were compared using Student’s t-test. Statistical significance was assumed at a value of *p < 0.05, **p < 0.01, ***p <0.001.

**Supplementary Tables**

**Table S1. A summary of reported representative hafnium-based nanomaterials for biological application.**

| **Materials** | **Functional components** | **Therapeutic modality** | **Engineering strategy** | **Sono-piezoelectric effects** | **Enzyme-mimic** | **Biological Effects** | **Reference** |
| --- | --- | --- | --- | --- | --- | --- | --- |
| HMO | Mn; HfO_2_ | Sono-piezoelectric & Enzymatic catalytic | Metal-doped  & Oxygen vacancies | **Yes** | POD/ CAT/GPx | PANoptosis  ICD | ***This work*** |
| TPU-HfO_2_ | HfO_2_ | SDT | no | no | no | Apoptosis | *ACS Nano*. **2024**, 18 (3), 2485-2499 |
| HfMnH | HfO_2_; MnO_2_ | RT/CDT | no | no | CAT/ GPx | cGAS-STING | *ACS Nano*. **2024**, 18 (5), 4189-4204 |
| SORT HfO_2_ | HfO_2_ | CDT | no | no | CAT/ SOD/ GPx | Inhibited apoptosis and inflammation | *Adv. Mater.* **2024**, 36, 2308098. |
| Hb@Hf-Ce6 | Hb; Ce6; PD-1 | RT/RDT/IT | Self-assembly | no | no | Induced apoptosis and effector T cells infiltration; Abscopal antitumor effect | *Adv. Sci*. **2021**, 8, 2003338. |
| Hf-nIm@PEG | Hf; 2-nIm | RT/ IT | Reversed-phase microemulsion | no | no | Apoptosis; ICD; Activated  cGAS-STING pathway | *Adv. Mater*. **2023**, 35, 2302220. |
| RPB7H | BPA771; CRGDK; HfO_2_ | RT/ RDT | PROTAC | no | NOX4 | Apoptosis; Downregulate BRD4-RAD51AP1 pathway | *Adv. Mater*. **2024**, 36, 2314132. |
| CBL@HfO_2_ | HfO_2_; CBL0137 | RT | Stöber's method | no | no | Augmented DSBs | *Adv. Mater.* **2024**, 36, 2313991. |
| HfO_2_ | HfO_2_ | RT | no | no | no | Treated tumor | *ACS Nano*. **2024**, 18 (33), 22378-22389. |
| HfO_2_ | HfO_2_ | RT | no | no | no | Enhanced cellular oxidative stress | *Nat Commun.* **2022**, 13, 3248. |
| HfO_2_ | HfO_2_ | RT | no | no | no | Induced abscopal antitumor effect; CD8^+^ T cells infiltration | *Journal of Clinical Oncology.* **2018**, 36, e15149-e15149 |
| NBTXR3 | HfO_2_ | RT | no | no | no | Increased the anti-PD1 and anti-CTLA4 efficacy | *Cancer Res.* **2019**, 79,  3225. |
| DAC@O-HONs | HfO_2_; DAC | RT/RDT | no | no | no | Induced apoptosis into pyroptosis | *Nano Today*. **2023**, 52,  101997. |
| HfO_2_ | HfO_2_ | RT/RDT | no | no | no | Apoptosis | *Biomaterials*. **2020**, 226,  119538. |
| M/H-D | HfO_2_; MoS_2_ | RT/CDT | Self-assembled coordination | no | POD | Apoptosis | *ACS Nano.* **2020**, 14, 8, 10001-10017. |

CDT is short for Chemodynamic therapy. RT is short for Radiotherapy. RDT is short for Radiodynamic therapy. IT is short for Immunotherapy.

**Table S2. A summary of reported representative piezoelectric biomaterials for cancer treatment.**

| **Materials** | **Bandgap (E_g_)** | | **Enzyme-Mimicking Activities** | **Biocompatibility**  **in Vitro** | **Biosafety Dose**  **in Vivo** | **Antitumor Effects** | **Reference** |
| --- | --- | --- | --- | --- | --- | --- | --- |
| HMO | 1.89 eV | | Km=1.622 mM (POD)  Km=4.101 mM (GPx) | L929: 250 μg/mL  HUVEC: 250 μg/mL  HEK-293: 250 μg/mL  RAW 264.7: 250 μg/mL | 25 mg/kg | PANoptosis  ICD | ***This work*** |
| BaTiO_3_ | 2.56 eV | | no | 4T1: 500 μg/mL | 2.5 mg/kg | no | *Adv. Mater.***2020**, 32, 2001976*.* |
| Bi_2_MoO_6_ | 2.97 eV | | no | L929: 200 μg/mL | 10 mg/kg | Apoptosis | *Adv. Mater.* **2021**, 33, 2106838. |
| NaNbO_3_ | 2.46 eV | | no | 4T1: 200 μg/mL | 2 mg/kg | Apoptosis | *Nano Energy.* **2021**, 79, 105485. |
| ZnO | 3.3 eV | | no | MCF-7: 20 μg/mL | 5 mg/kg | Apoptosis | *Chemical Engineering Journal*. **2022**, 435, 135039. |
| SnS | 1.81 eV | | no | HeLa: 200 μg/mL | 2 mg/kg | Apoptosis | *J. Mater. Chem. A*. **2023**, 11, 7331-7343.窗体顶端 |
| MoS_2_ | 1.9 eV | | no | KB: 50 μg/mL | 2 mg/kg | no | *Bioactive Materials*. **2021**, 6 (11), 4209-4242. |
| B-BTO | 1.9 eV | | no | 3T3: 100 μg/mL | 10mg/kg | Apoptosis | *Adv. Funct. Mater.* **2025**, 2412983. |
| Bi@BTO | 3.26 eV | | no | MSCs: 400 μg/mL | 2.5 mg/kg | Apoptosis | *Chemical Engineering Journal*. **2022**, 442 (2), 136380. |
| RuNC/BTO | no | | Km=25.77 mM (POD) | no | 10 mg/kg | Apoptosis | *Small.* **2023**, 19, 2206911. |
| SBN/SNO | 3.57 eV | | no | HUVEC: 200 μg/mL | 20 mg/kg | Apoptosis | *Adv. Funct. Mater.***2024**, 34, 2405929. |
| Mn-ZnO | 2.95 eV | | Km=25.9 mM (POD) | L929: 300 μg/mL | 15 mg/kg | Ferroptosis | *Adv. Mater.***2023**, 35, 2304262. |
| BCO-V_Cu_ | 1.67 eV | | Km=40.36 mM (POD) | L929: 200 μg/mL | no | Cuproptosis  Apoptosis  ICD | *Adv. Mater.***2024**, 36, 2403253. |
| Cu-NS SA | no | Km=0.45 mM (POD)  Km=0.33 mM (GSHOx) | | L929: 200 μg/mL | no | Pyroptosis  ICD | *Adv. Mater.***2024**, 36, 2312124. |
| TiO2-Ru-PEG | 2.26 eV | | no | MB49: 28 μg/mL  SV-HUC-1: 28 μg/mL | 10 mg/kg | Apoptosis | *Adv. Mater.***2024**, 36, 2401252. |

**Supplementary Figures and Caption**





**Figure S1.** (**a**) TEM images of HMO. (**b**) Zeta potentials of HMO in different solutions (n = 3). (**c**) Hydrodynamic diameters of HMO in different solutions at 0, 4, 8, 12, 24, and 48 h (n = 3). (**d**) Hydrodynamic diameters of HMO in different media. Data are presented as mean ± standard deviation (S.D.).


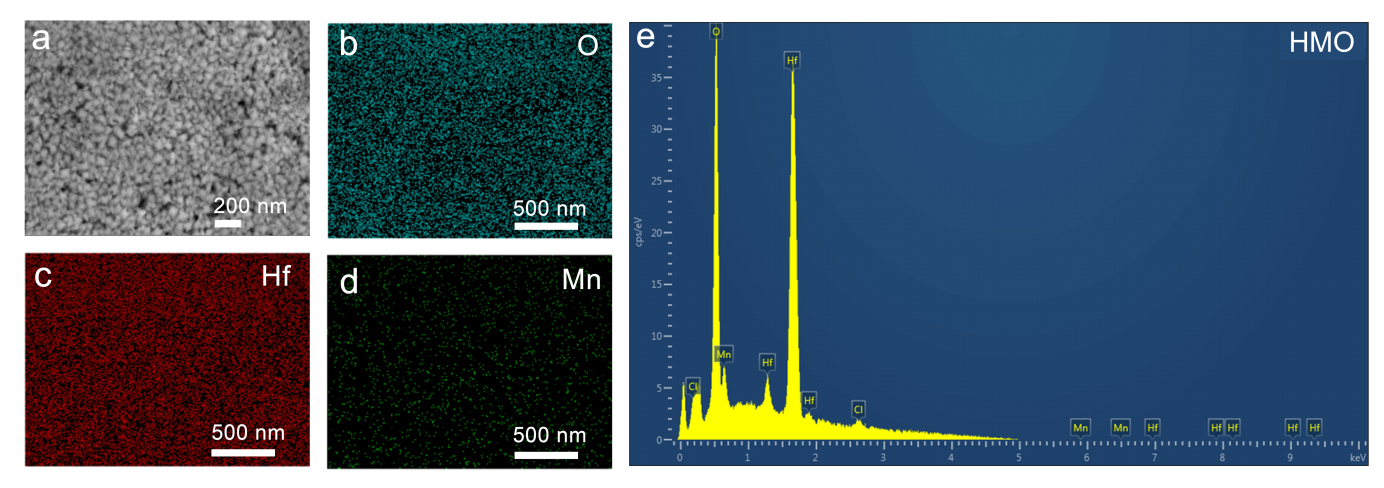


**Figure S2.** (**a**) SEM image of HMO. (**b-d**) Elemental mapping images of Hf, Mn, O in HMO. (**e**) Energy dispersive spectroscopy (EDS) profile of HMO.


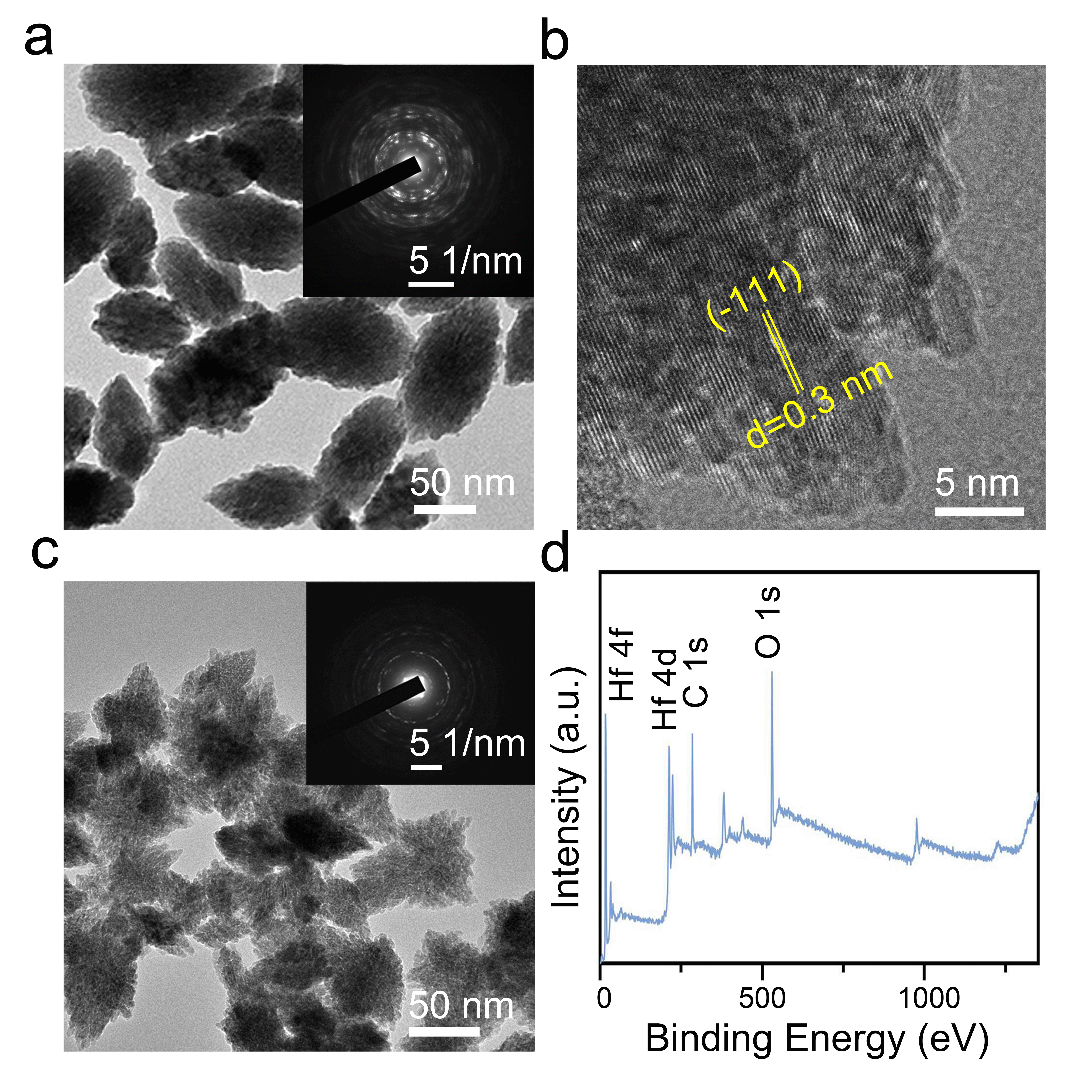


**Figure S3.** (**a**) TEM, (**b**) high-resolution TEM (HRTEM) images of HfO_2_. (**c**) TEM image of HMO. (**d**) XPS spectra of HfO_2_. Inset shows the selected area electron diffraction (SAED) profile of HfO_2_ and HMO, respectively.


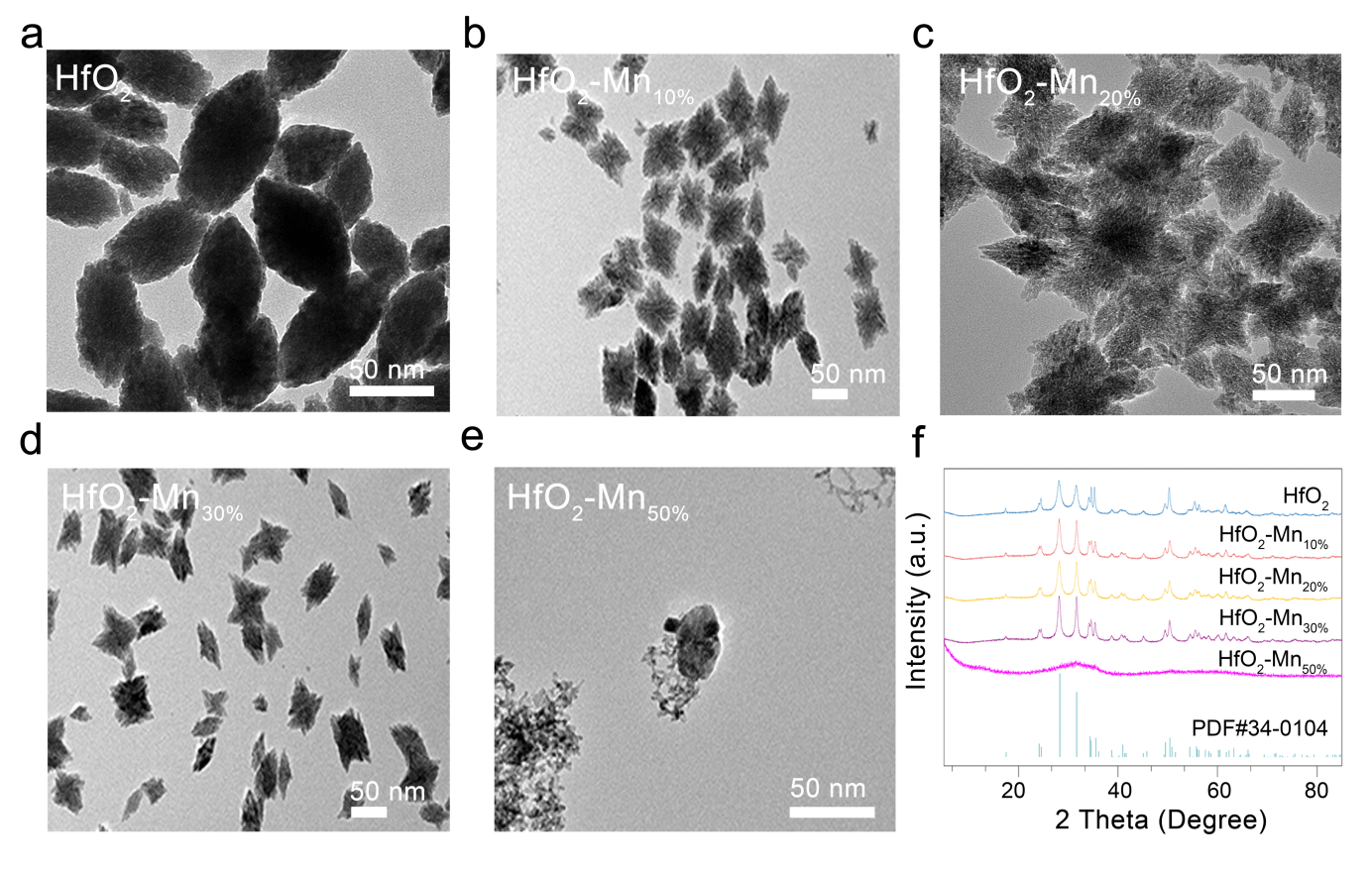


**Figure S4.** (**a-e**) TEM images of HfO_2_ and Mn-doped HfO_2_ with different Mn contents. (**f**) XRD of HfO_2_ and Mn-doped HfO_2_ with different Mn contents.


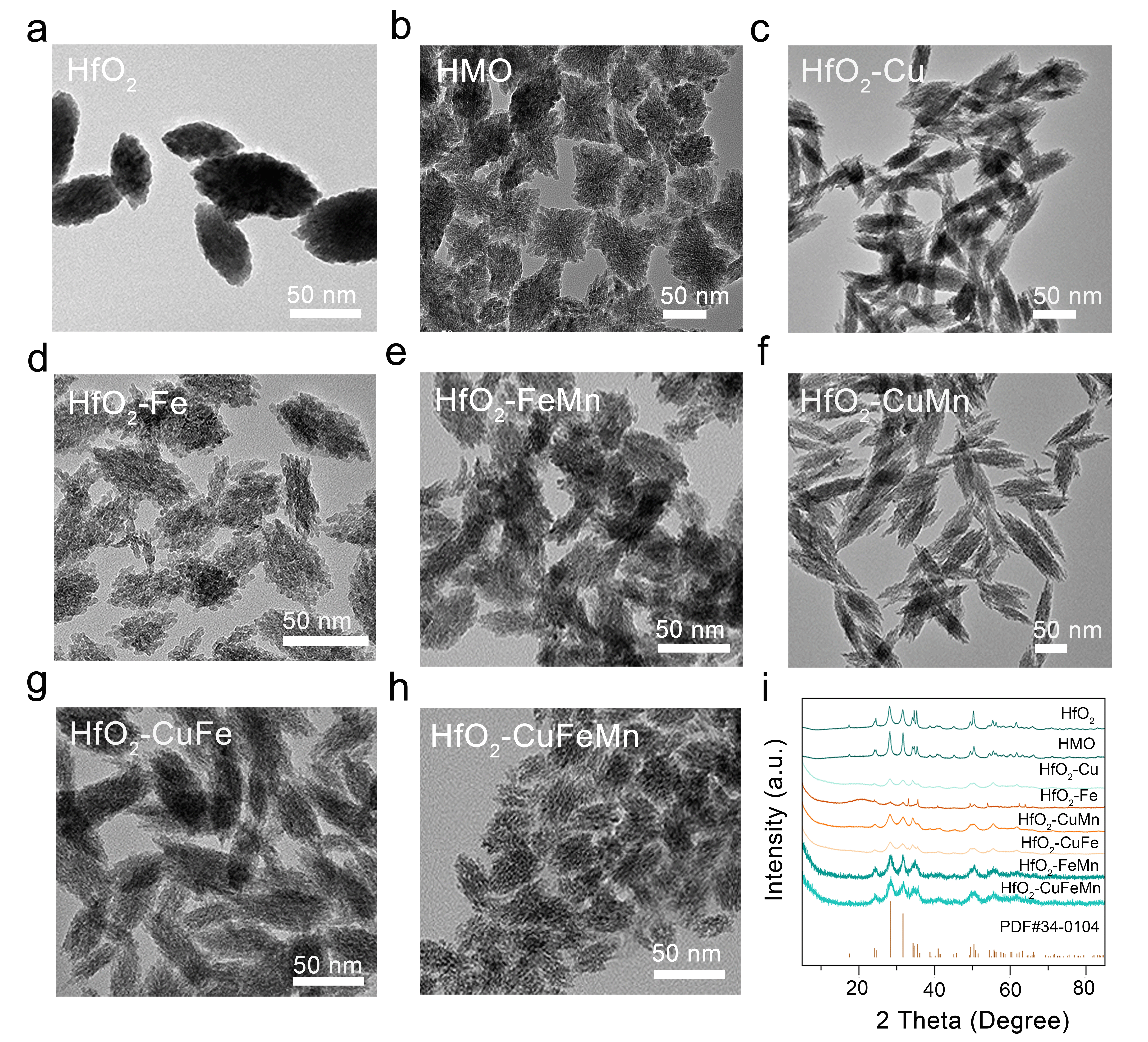


**Figure S5.** (**a**) TEM images of HfO_2_ and (**b-h**) different metal ions doped HfO_2_. (**i**) XRD of HfO_2_ and Mn-doped HfO_2_ with different Mn contents.

**Figure S6.** TEM image and elemental mapping of (**a**) Hf of HfO_2_, (**b**) Hf, Cu of HfO_2_-Cu, (**c**) Mn of HMO, (**d**) Fe of HfO_2_-Fe, (**e**) Hf, Cu, Mn and O of HfO_2_-CuMn, (**f**) Hf, Cu, Fe and O of HfO_2_-CuMn, (**g**) Hf, Fe, Mn and O of HfO_2_-FeMn, (**h**) Hf, Cu, Fe, Mn and O of HfO_2_-CuFeMn.


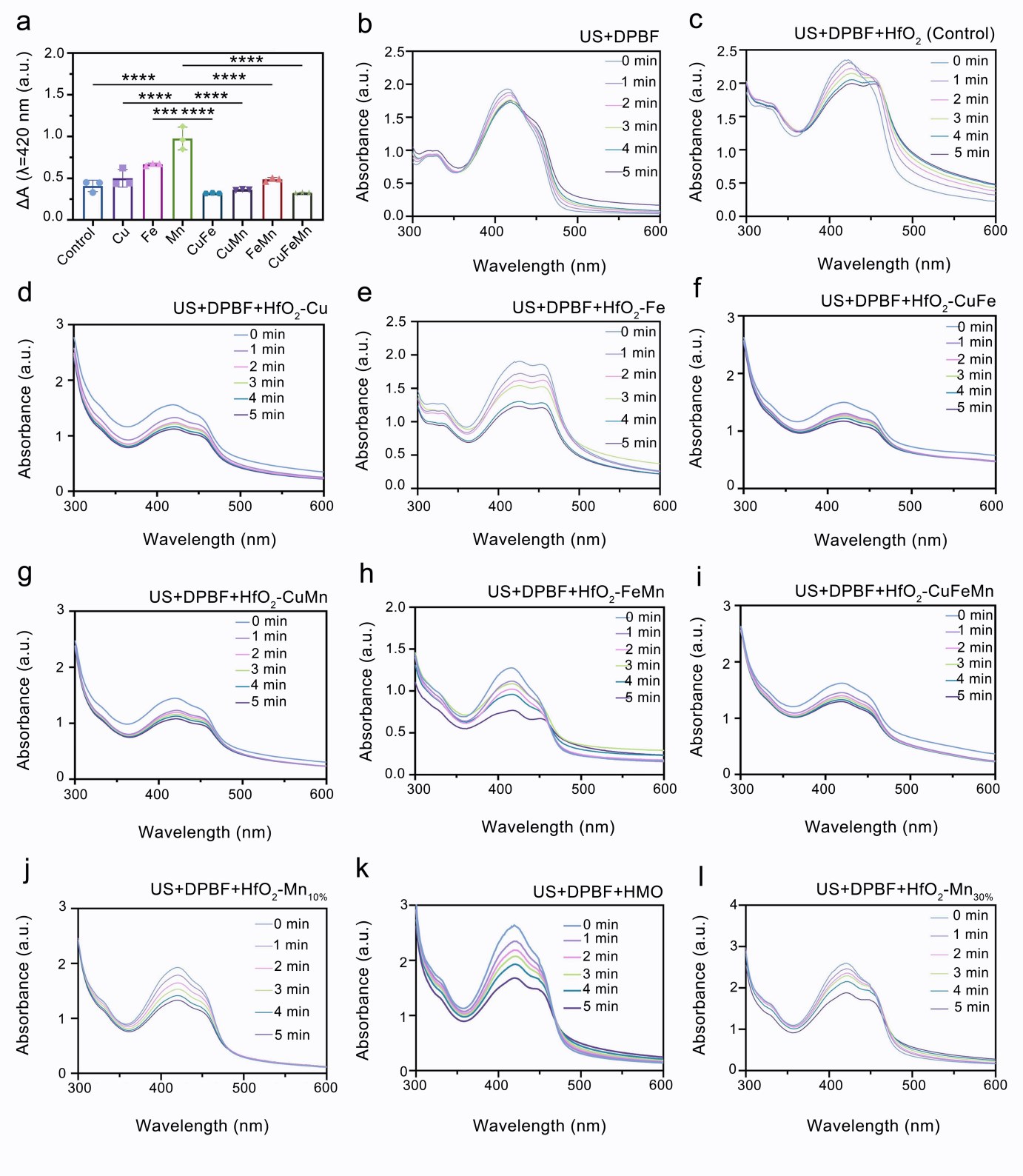


**Figure S7.** (**a**) UV-vis absorbance changes of DPBF at 420 nm after incubated with the different metal-ion doped HfO_2_ at different Mn-doping content under US excitation. Data are presented as mean ± standard deviation (S.D.) (n = 3). (**b-i**) US-triggered (1.0 MHz, 1.0 W/cm^2^, 50% cycle) ^1^O_2_ generation catalyzed with the different metal-ion doped HfO_2_. (**j-l**) US-triggered (1.0 MHz, 1.0 W/cm^2^, 50% cycle) ^1^O_2_ generation catalyzed with the Mn-substituted HfO_2_ at different Mn-doping content under US excitation.

**Figure S8.** Tauc plots of (**a**) HfO_2_, and (**b**) HMO.


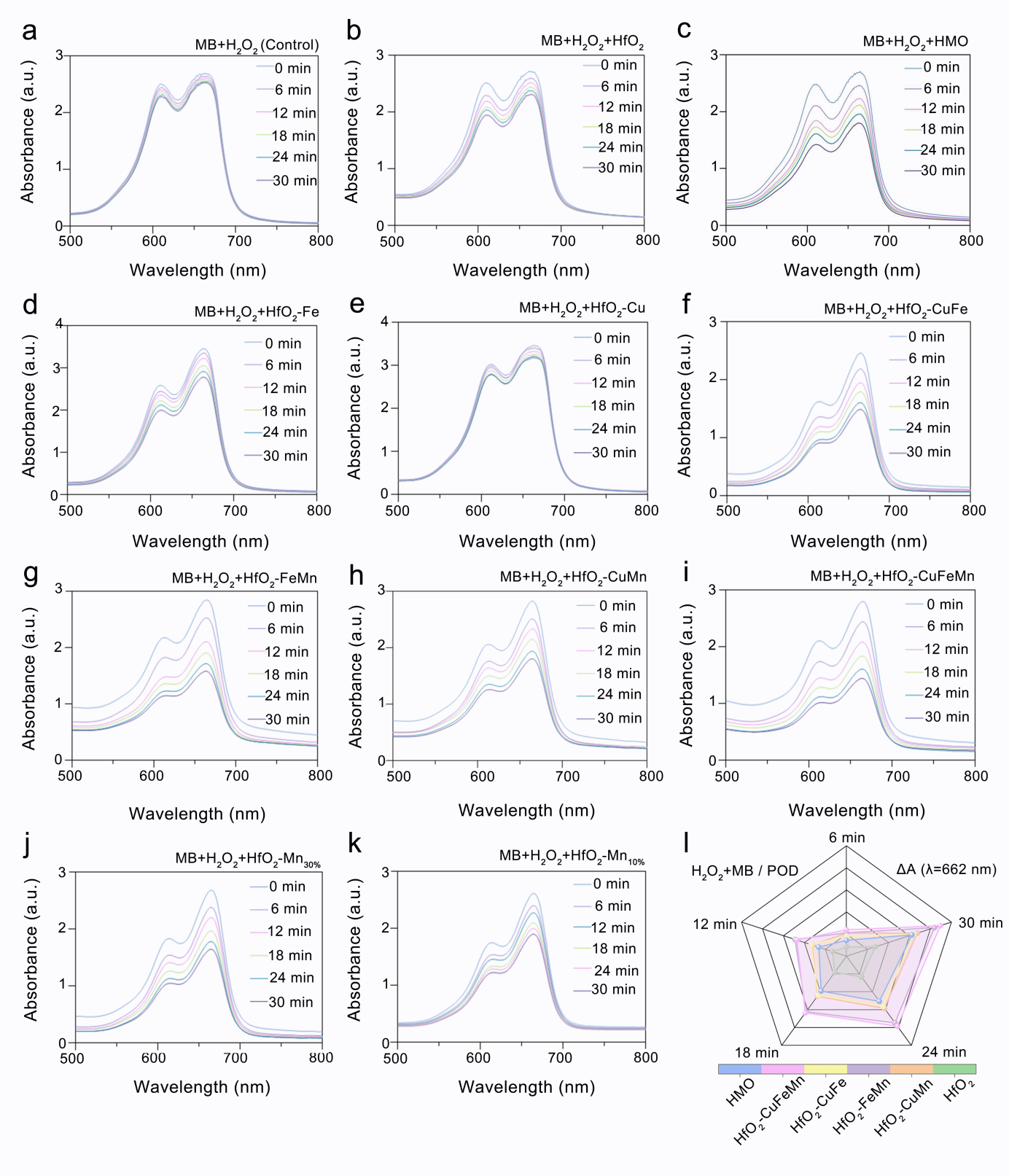


**Figure. S9** (**a-i**) UV-vis absorbance of MB treated with different metal ions doped HfO_2_ containing H_2_O_2_ as a substrate at different times. (**j-k**) UV-vis absorbance of MB and H_2_O_2_ exposed to HfO_2_-Mn with different Mn-doping contents at different times. (**l**) UV-vis absorbance changes of MB at 662 nm treated with different metal-ion doped HfO_2_ containing H_2_O_2_ as a substrate at different times.


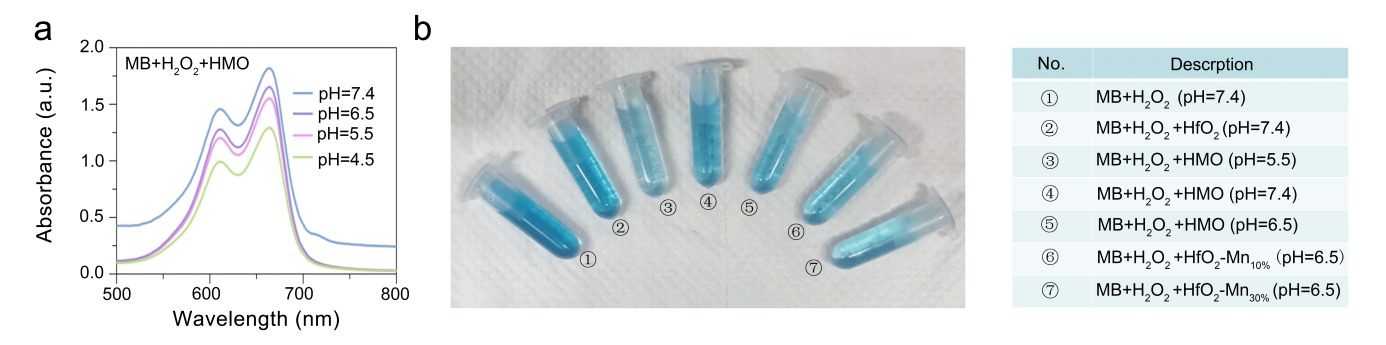


**Figure S10.** (**a**) UV-vis absorbance changes of HMO incubated with H_2_O_2_ using MB as a ·OH probe at different pH (7.4, 6.5, 5.5, and 4.5). (**b**) The photographs of resulting MB solutions after different treatments.


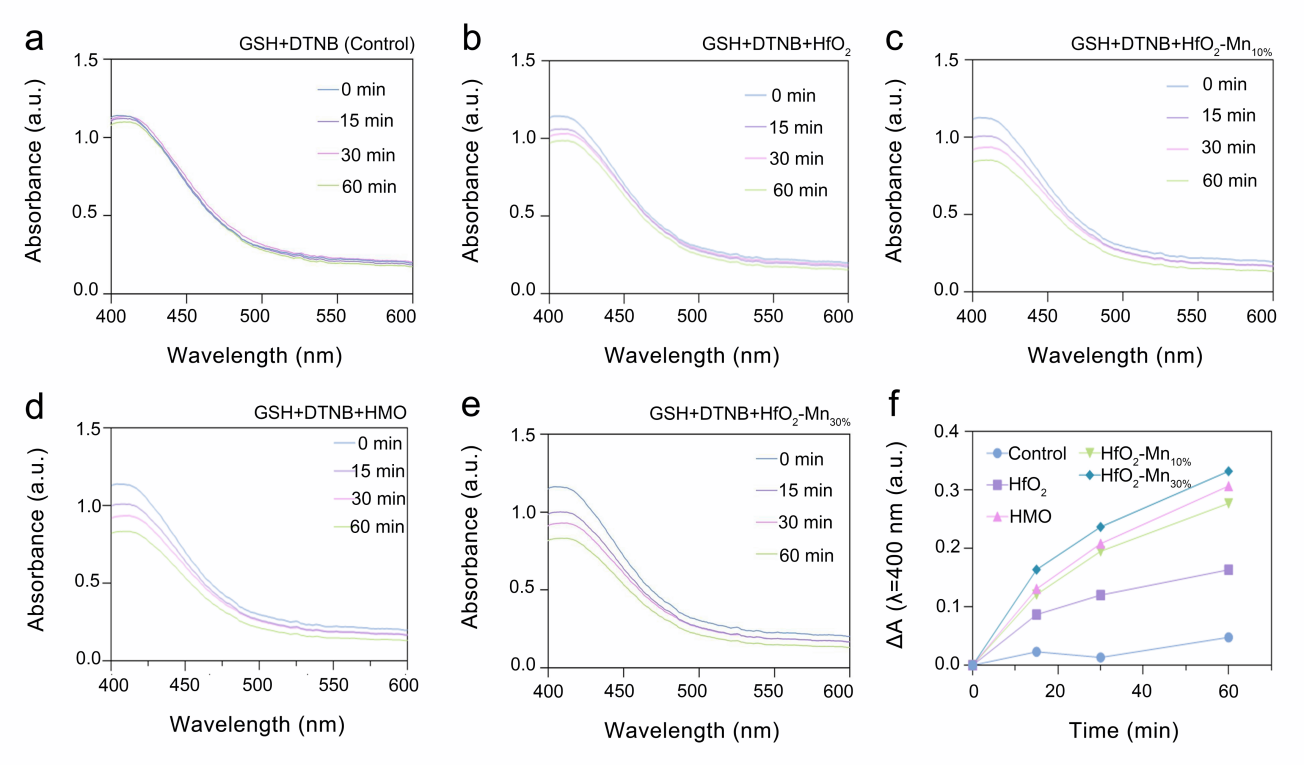


**Figure S11.** (**a-e**) UV-vis absorbance change of DTNB treated with GSH and HfO_2_-Mn with different Mn-doping contents. (**f**) UV-vis absorbance change of DTNB treated with GSH and HfO_2_-Mn with different Mn-doping contents under the excitation of 400 nm by GSH as a substrate at different time.

**
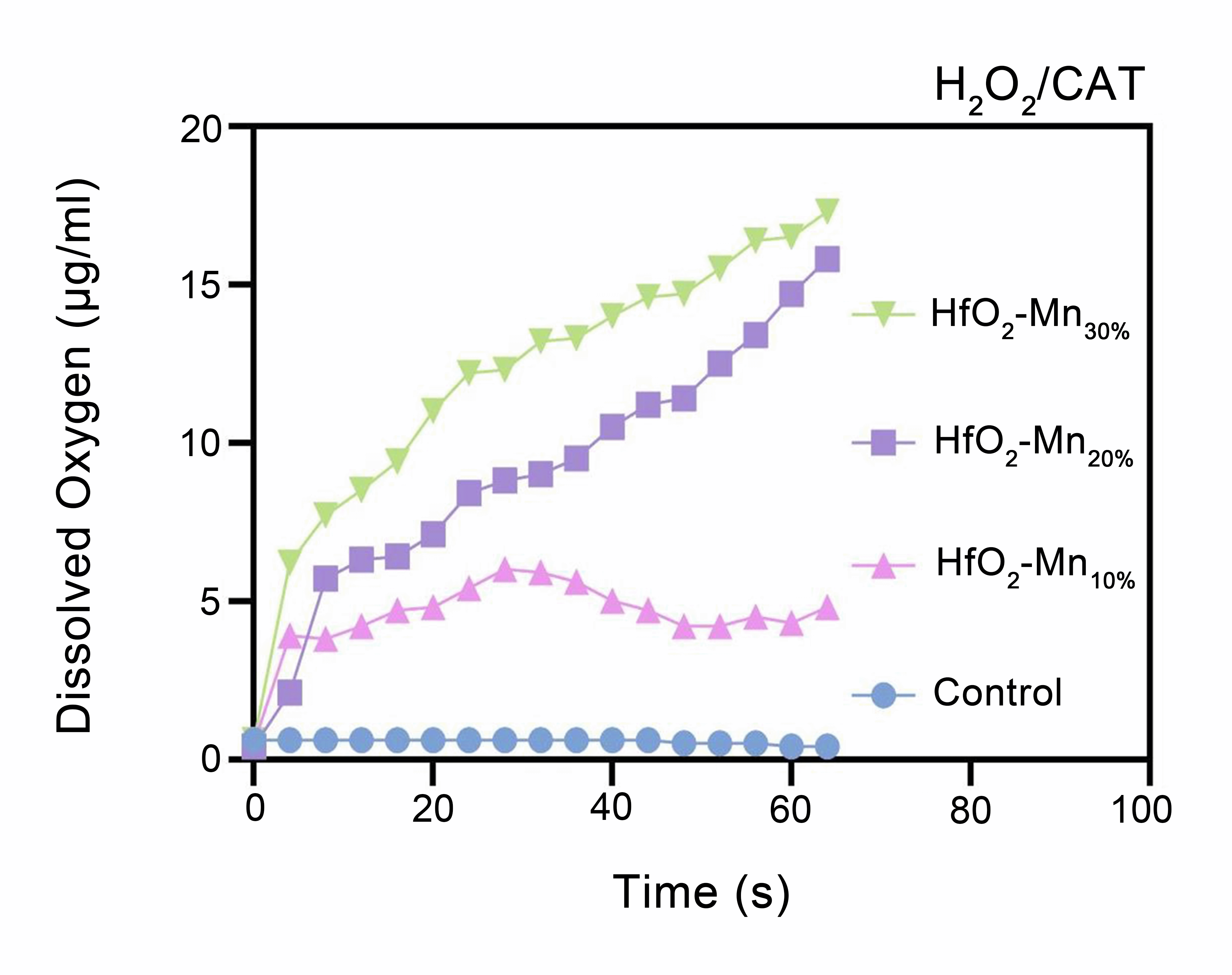
**

**Figure S12.** Dissolved oxygen of H_2_O_2_ incubated with HfO_2_-Mn at different Mn-doping contents.


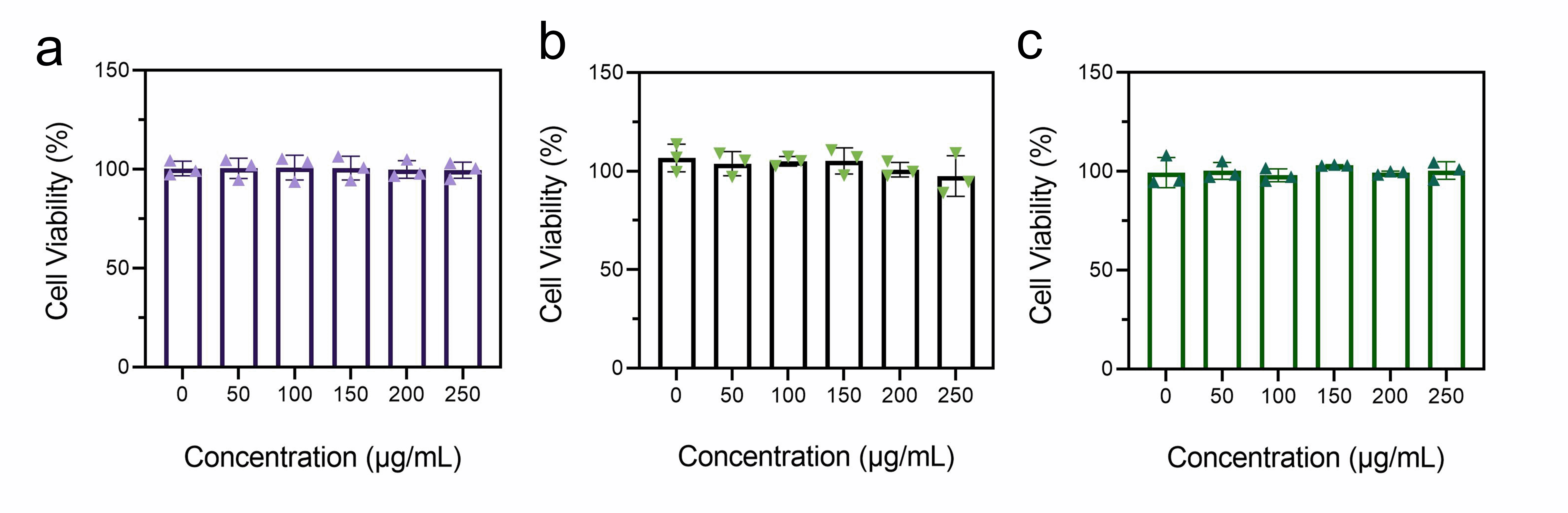


**Figure S13.** Cell viability of (**a**) mouse fibroblast (L-929), (**b**) human embryonic kidney 293 (HEK-293), and (**c**) RAW 264.7 cells incubated with different HMO concentrations for 24 h (n = 3). Data are presented as mean ± standard deviation (S.D.).


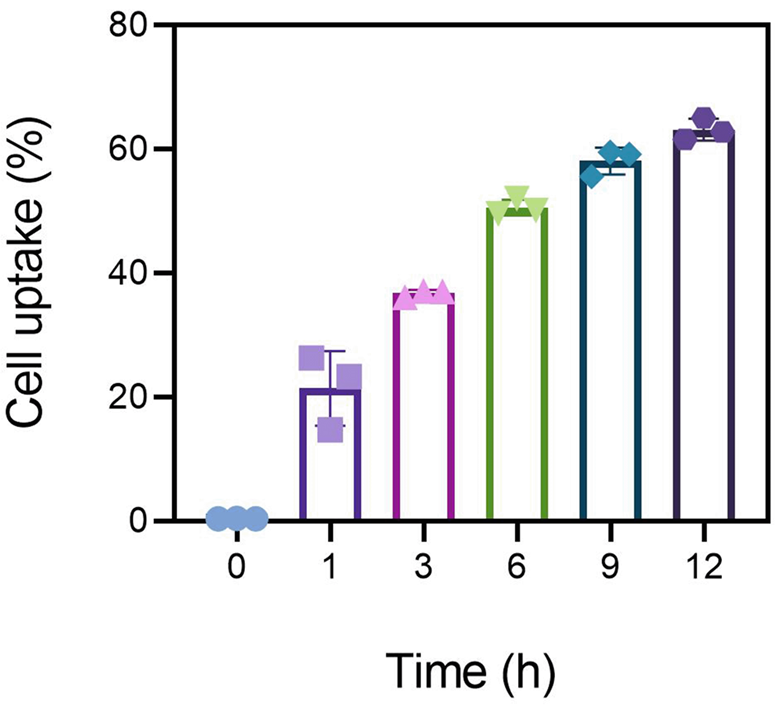


**Figure S14.** FCM analysis of cellular uptake behavior of FITC-labeled HMO in 4T1 cells at different time intervals (n = 3).


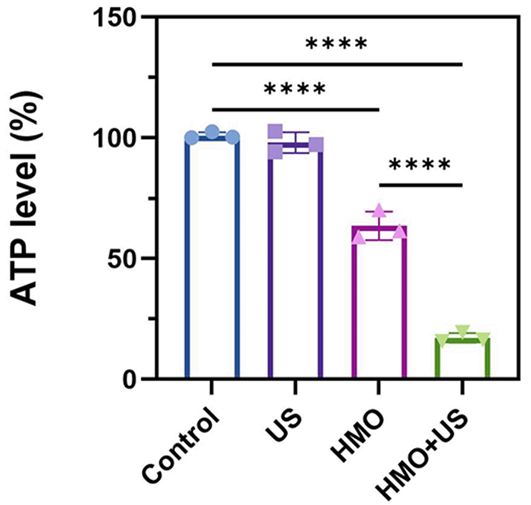


**Figure S15**. (**a**) ATP levels in 4T1 cells after different treatments (n = 3). Data are presented as mean ± standard deviation (S.D.).


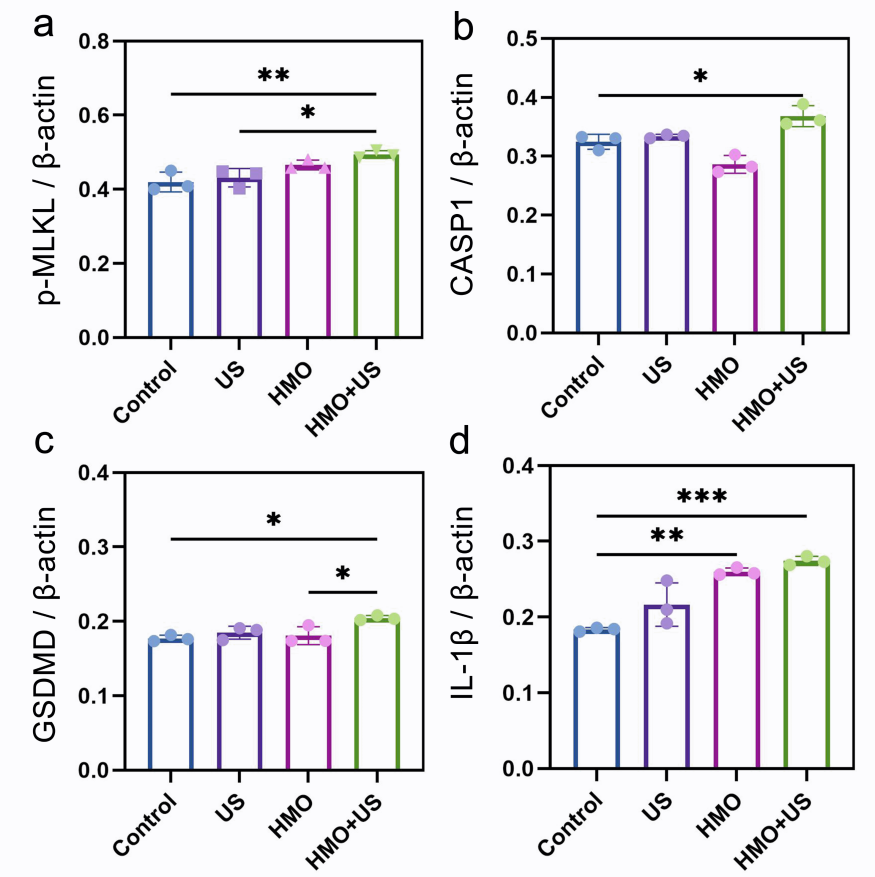


**Figure S16.** WB analysis of p-MLKL, Caspase-1 (CASP1), GSDMD, and IL-1β expression (n=3). Data are presented as mean ± standard deviation (S.D.).


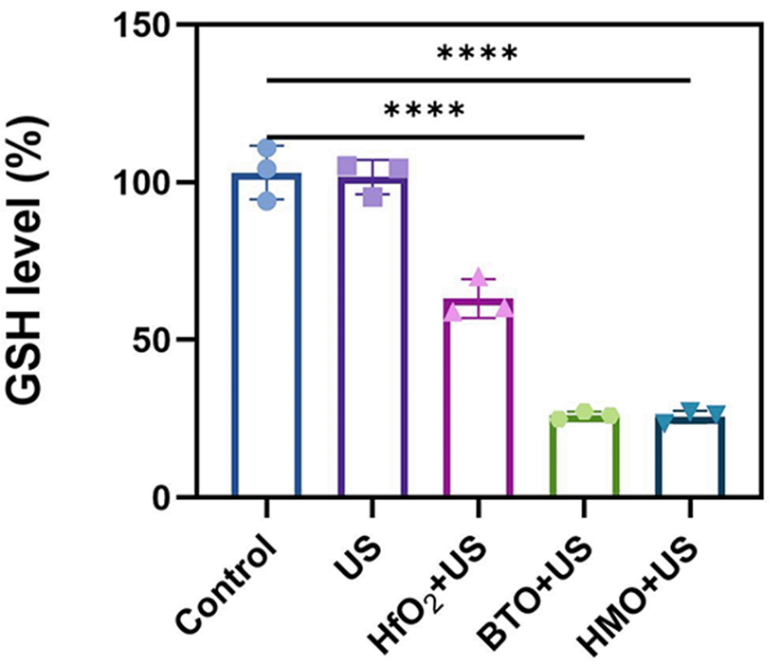


**Figure S17**. GSH levels in 4T1 cells after different treatments (n = 3). Data are presented as mean ± standard deviation (S.D.)


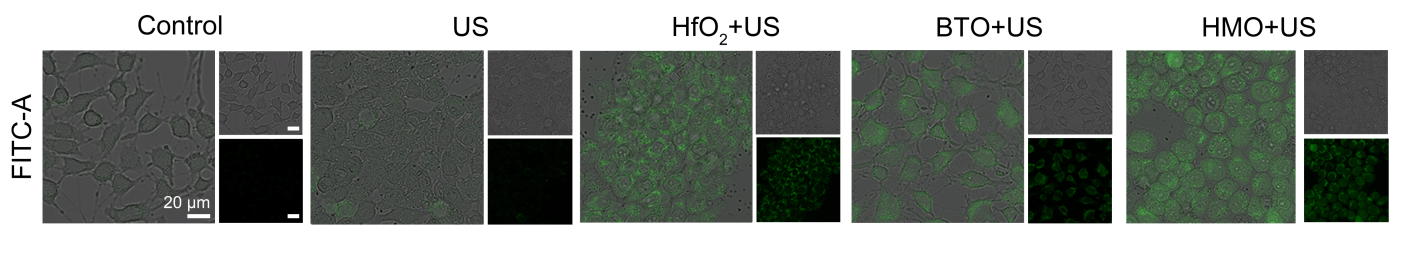


**Figure S18**. CLSM images of ROS generation in 4T1 cells with various treatments using DCFH-DA as a probe.


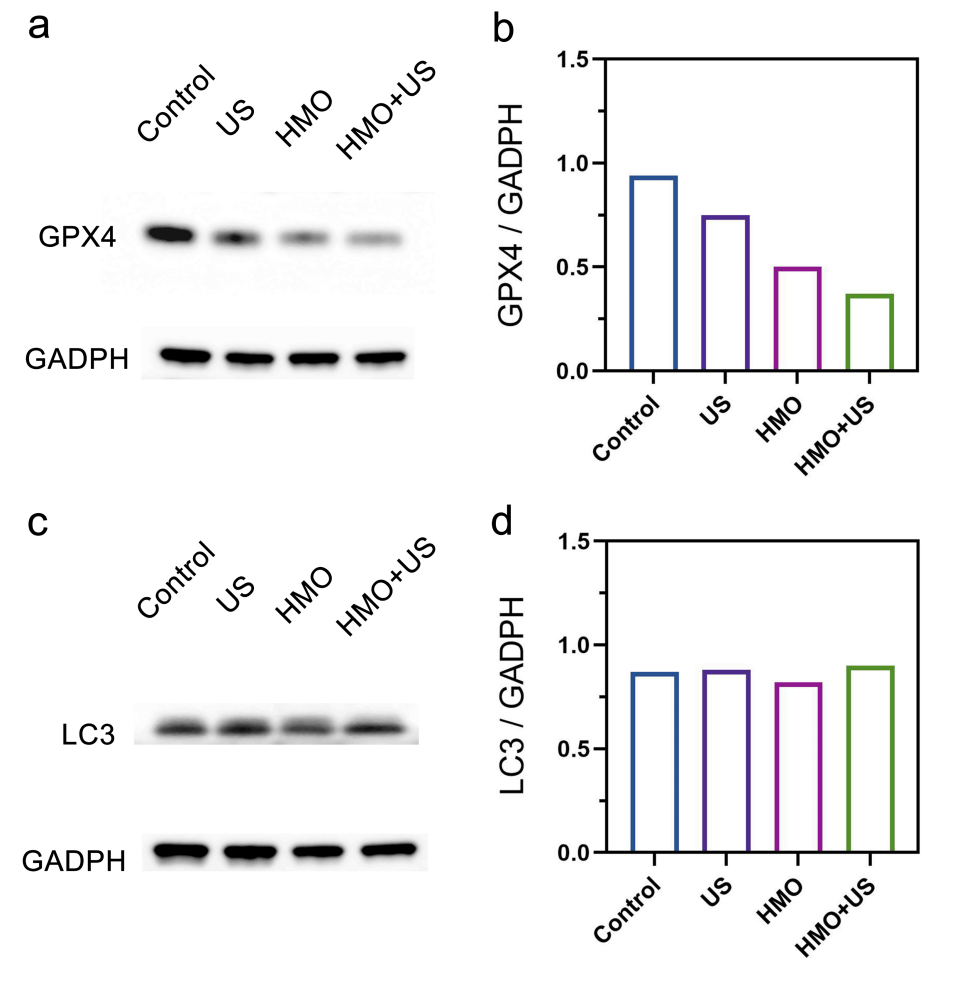


**Figure S19.** WB analysis of (a, b) GPX4 and (c, d) LC3 expression in 4T1 cells after different treatments.


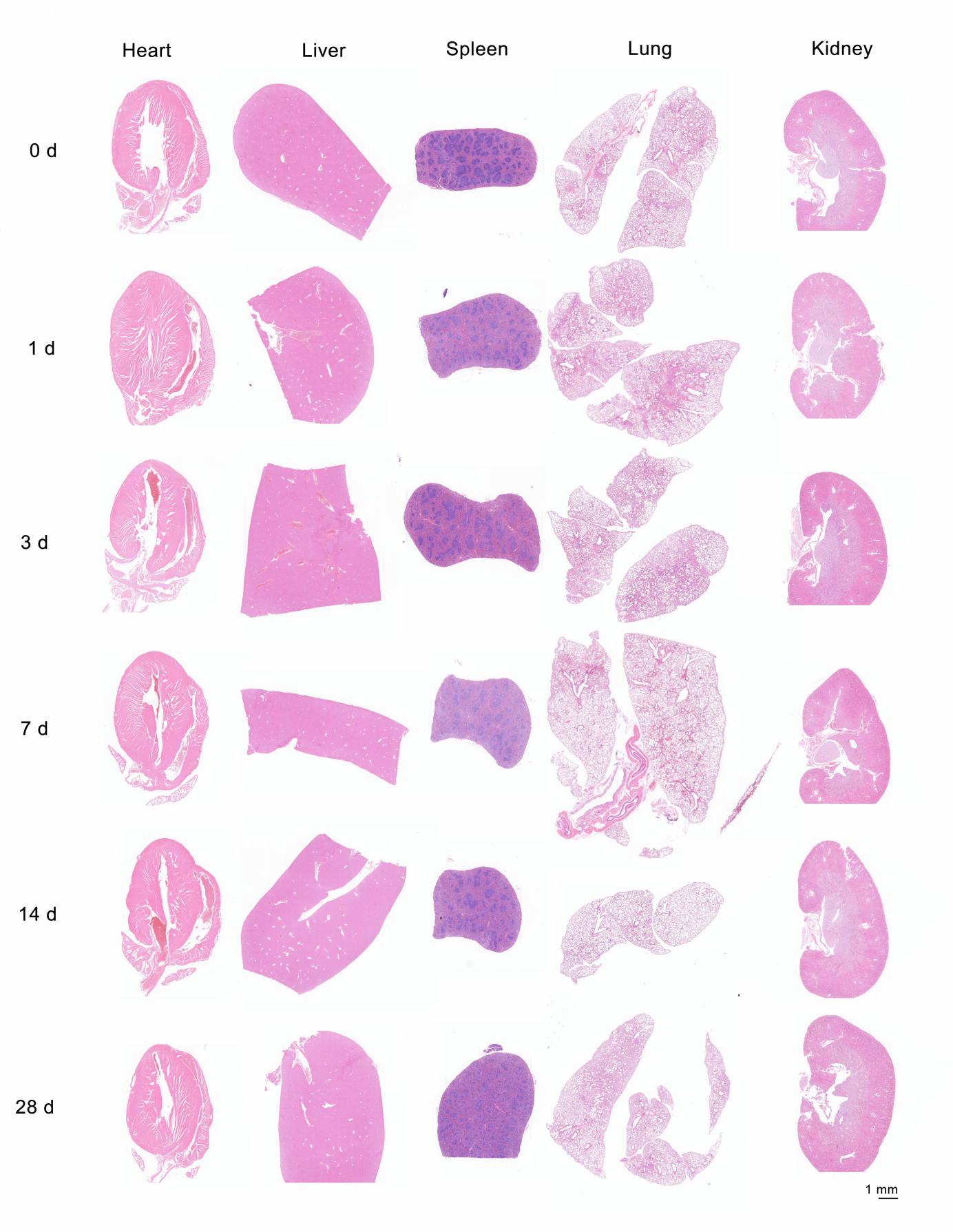


**Figure** **S20.** H&E staining of major organs (heart, liver, kidney, lung, and spleen) in the healthy mice injected with HMO at different time intervals.


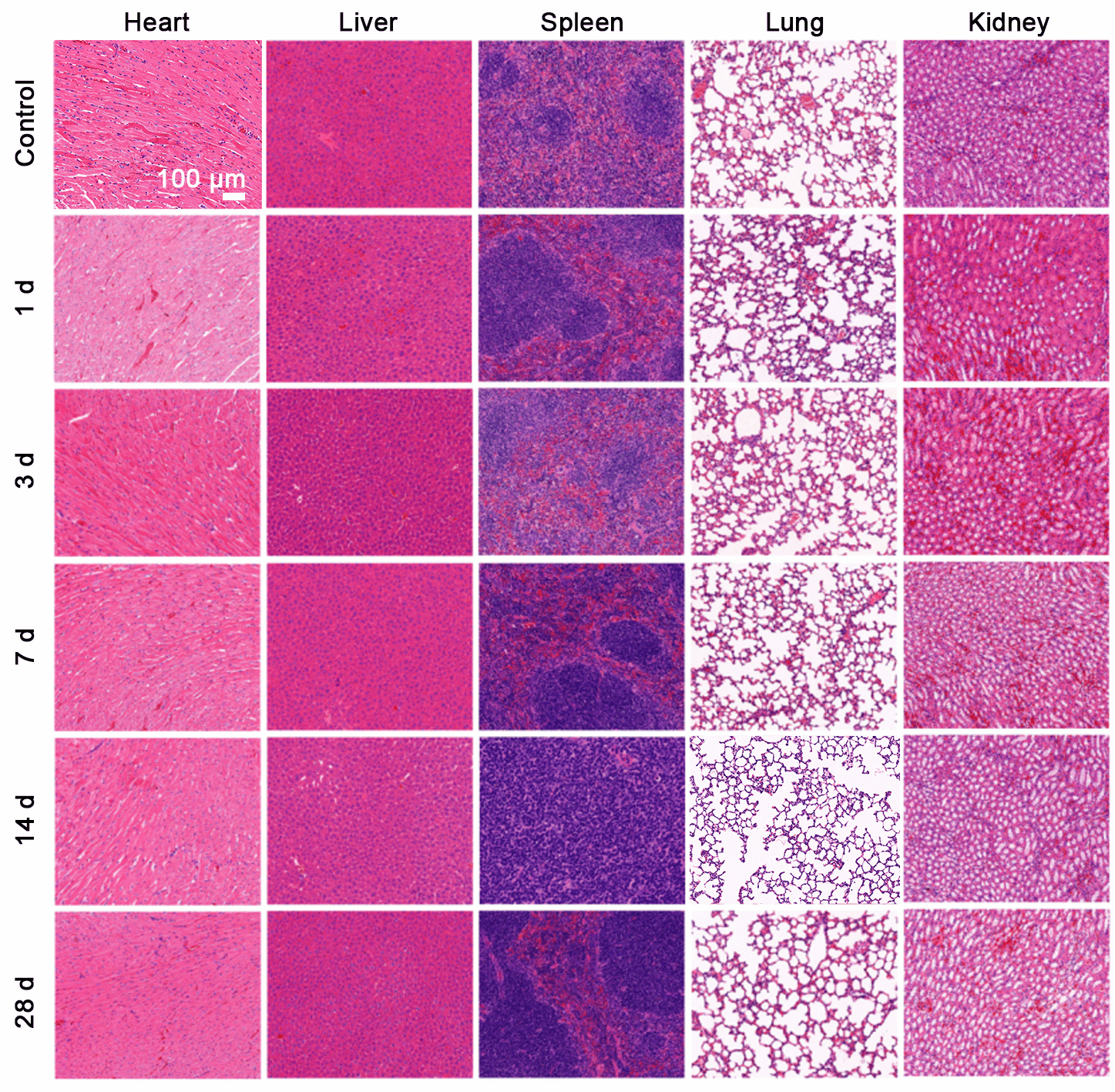


**Figure S21**. H&E staining of major organs in the healthy mice injected with HMO at different time intervals.


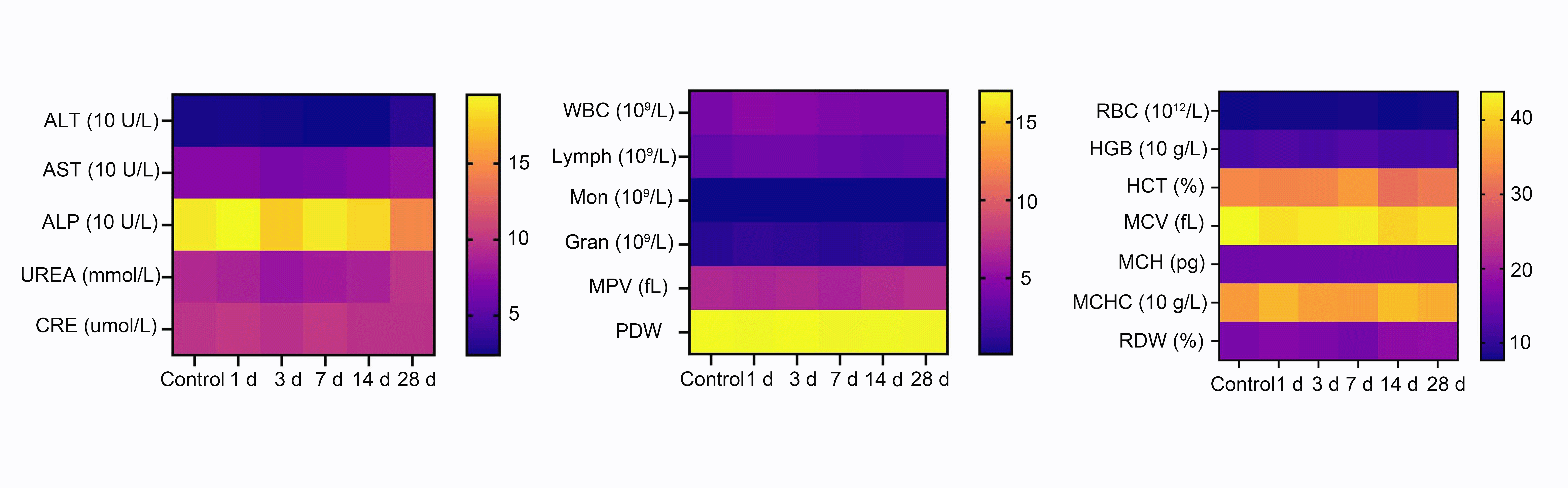


**Figure S22.** Blood biochemistry and hematological parameters of the healthy mice injected with HMO at different time intervals.
